# Genome-wide analysis of dirigent gene family in pepper (*Capsicum annuum* L.) and characterization of *CaDIR7* in biotic and abiotic stresses

**DOI:** 10.1038/s41598-018-23761-0

**Published:** 2018-04-03

**Authors:** Abid Khan, Ru-Jian Li, Jian-Tian Sun, Fang Ma, Huai-Xia Zhang, Jing-Hao Jin, Muhammad Ali, Saeed ul Haq, Jun-E Wang, Zhen-Hui Gong

**Affiliations:** 1 College of Horticulture, Northwest A&F University, Yangling, Shaanxi 712100 P. R. China; 2 College of Horticulture, Shanxi Agricultural University, Taigu, 030801 Shanxi China

## Abstract

The dirigent (DIR and DIR-like) proteins involved in lignification, play a pivotal role against biotic and abiotic stresses in plants. However, no information is available about DIR gene family in pepper (*Capsicum annuum* L.). In this study, 24 putative dirigent genes (CaDIRs) were identified, their gene structure, genome location, gene duplication and phylogenetic relationship were elucidated. Tissue-specific expression analysis displayed the highest transcription levels in flower, stem and leaf. Some CaDIRs were up-regulated by virulent (*CaDIR2*, *3*, *6*, *7*, *11*, *14*, *16*, *22* and *23*) and avirulent (*CaDIR3*, *5*, *7*, *16*, *20*, *22*, *23* and *24*) *Phytophthora capsici* strains, as well as by Methyl jasmonate, salicylic acid, NaCl and mannitol stresses. Acid-soluble lignin content increased (103.21%) after *P*. *capsici* inoculation (48-hour). Silencing of *CaDIR7* weakened plant defense by reducing (~50%) root activity and made plants more susceptible (35.7%) to *P*. *capsici* and NaCl (300 mM). Leaf discs of the *CaDIR7*:silenced plants exposed to NaCl and mannitol (300 mM each), exhibited a significant decrease (56.25% and 48% respectively) in the chlorophyll content. These results suggested that *CaDIR7* is involved in pepper defense response against pathogen and abiotic stresses and the study will provide basic insights for future research regarding CaDIRs.

## Introduction

Pepper (*Capsicum annuum* L.) is an important solanaceous vegetable worldwide. Many vegetables are affected by the oomycete pathogen *P*. *capsici*, which has been reported to infest pepper, eggplant, tomato, all cucurbits, and more recently snap and limabeans^1,2^. In general, with a sessile and autotrophic lifestyle, plants grown in the open environment are always vulnerable to various biotic and abiotic stresses, such as pathogen infections, pest attacks, extreme temperatures, drought, salinity, and heavy metals^3,4^. These stresses cause a significant reduction in yield and quality. In response, plants have evolved some sophisticated defense mechanisms, including oxidative burst, the deposition of lignin and callose into the cell wall and regulation of signaling networks, to combat these stresses^5–8^. To control pathogen growth and invasion in the host tissues, inducible biochemical reactions create a protective physiological condition^9^. The inducible defense responses of plant include the synthesis of signaling molecules such as salicylic acid, ethylene and jasmonates, which in turn regulate gene expression and produce defense molecules such as reactive oxygen species (ROS), phenylpropanoids, phytoalexins and pathogenesis-related (PR)^10^. In response to biotic and abiotic challenges, plants activate a variety of genes, including the DIR gene^9,11^. DIR is a disease resistance responsive gene (DRRG), which enhances the stress resistance in different crop plants^11^.

The word dirigent comes from the Latin word *dirigere* (to guide or align), and dirigent proteins were found for the first time in *Forsythia intermedia*^12^. DIR proteins from *Forsythia suspensa*^13^, *Podophyllum peltatum*^14^, and *Thuja plicata* (western red cedar)^15^ are biochemically involved in directing the stereo-specific coupling of E-coniferyl alcohol to produce the lignan ( + )-pinoresinol. Based on this activity and its associated cellular localization in *F*. *intermedia*, DIR proteins could also function in the formation of lignin^16^. If DIR proteins are absent, then at the 8–8′, 8–5′, or 8-O-4′ positions a non-specific radical-radical coupling occurs, resulting in racemic lignan products^13,17^. DIR proteins share a dirigent-conserved domain with disease resistance response (DRR) family proteins^18^, and they are believed to mediate the free radical coupling of monolignol plant phenols in plants to yield lignans and lignins^13,16^; thus, DIRs have been culpably involved in disease resistance responses^19,20^. Lignan, either constitutive or inducible, has antifungal properties, which seems to be primarily involved in plant defense^21^. In defense response against pathogen infection, lignin deposition is considered to function as a physical barrier^22^. Moura *et al*.^23^ suggested that an increase in lignification occurs in response to pathogen attacks. Fang *et al*.^24^ noted that in mature leaves the thickened structure contains lignin, which acts as an effective physical barrier to pathogen attacks. As a non-degradable mechanical barrier for most microorganisms, lignin makes the host less susceptible. Lignin is an imperative compound that is primarily deposited in terminally differentiated cells of supportive and water-conducting tissues, and it is mainly associated with mechanical support, water transport in the xylem vessels and defense against pests and microorganisms^25^.

Earlier studies have shown that DIR proteins exist in almost all vascular plants, including lichens, ferns, gymnosperms and angiosperms^11,26–29^. In canola, when the DIR gene was expressed constitutively, increased resistance was observed against a broad range of fungal pathogens including *Rhizoctonia solani* and *Leptosphaeria maculans*^19^. The *BhDIR1* transcripts in *Boea hygrometrica* were found to be expressed in response to various abiotic stresses including dehydration, CaCl_2_, ABA, H_2_O_2_, EGTA, and temperature stresses^28^. Ralph and colleagues suggested that DIR proteins can be further divided into five subgroups, i.e., DIR-a, DIR-b, DIR-c, DIR-d and DIR-e^11^. A further increase in the number of DIR proteins and the appearance of two other subfamilies i.e. DIR-f and DIR-g, the DIR-b and DIR-d subfamilies were clustered together^27^.

Understanding the functions of the dirigent gene family in biological and physiological process would be a possible practicable approach to analyzing and improving the defense response of crops against biotic and abiotic stresses. However, no study has been conducted on the DIR gene family in pepper until now. Hence, the present study was designed to explore the DIR and DIR-like protein (CaDIRs) family in pepper through a genome-wide identification and gene expression analysis. A total of 24 CaDIRs from the pepper genome were identified through a bioinformatics analysis and PCR testing. Subsequently, we also performed a detailed analysis of the gene structure, conserved domains, tertiary structures, chromosomal distribution, gene duplication, cis-acting elements in the promoter regions and phylogenetic relationships to explore the evolutionary history of DIR and DIR-like protein expansion in pepper. In addition to ascertaining the probable role of CaDIRs in pepper, the expression patterns of CaDIRs in different plant tissues were evaluated, as well as their responses to *P*. *capsici* and various abiotic and hormonal stresses. Furthermore, the acid-soluble lignin content after *P*. *capsici* inoculation was also measured to confirm the role of CaDIRs in lignin biosynthesis. The function of the *CaDIR7* gene in pepper plants against *P*. *capsici* and salt stress was characterized through VIGS. The current study will provide a basis for the role of CaDIRs in pepper defense response against *P*. *capsici* and abiotic stresses and future insights for research on the DIR gene family.

## Materials and Methods

### Identification and Annotation of CaDIRs in Pepper

For identification of the CaDIR domain with accession no. PF03018, the methodology of our previous study was followed^30^. The alignment of the candidate CaDIRs from CM334 and Zunla-1 databases were conducted by DNAMAN software (Version 5.0) to select genes with differences in sequences in the two databases^31^. Gene-specific primer pairs **(**Supplementary Table S1) were designed by Primer Premier 5.0 (Premier Biosoft International, CA, U.S.A.) to amplify the different target regions, which were then aligned with sequences of the same gene from the CM334 and Zunla-1 databases to confirm the reliable sequences. Nomenclature of the putative CaDIRs was assigned based on their chromosome orders.

### Bioinformatics analysis, exon/intron structure determination and phylogenetic tree construction

Multiple sequence alignments of amino acid sequences were performed using DNAMAN software (Version 5.0). To compute the molecular formula, the total number of items, instability index, protein molecular weight (MW) and theoretical isoelectric point (*p*I), the amino acid sequences were blast in Expasy ProtoParam (http://web.expasy.org/protparam/). WoLF PSORT II (http://www.genscript.com/wolf-psort.html)^32^ and TargetP (http://www.cbs.dtu.dk/services/TargetP/)^33^ were used to predict the subcellular locations. N-glycosylation sites (Asn) of the CaDIRs were searched online using NetNGlyc 1.0 server (http://www.cbs.dtu.dk/services/NetNGlyc/)^34^. For phylogenetic tree construction, the amino acid sequences of DIR and DIR-like genes from different plant species were aligned using CLUSTALW as described by Guo *et al*.^35^ while MEGA 6.0 was used for tree construction^36^. The exon/intron structures of the CaDIRs were searched and presented as described by Kang *et al*.^37^. Prediction of the tertiary structures and homologs of CaDIRs was conducted through the online server Phyre2 (http://www.sbg.bio.ic.ac.uk/phyre2/html/page.cgi?id = index)^38^ as described by Li *et al*.^29^.

### Chromosomal Location and Duplication Analysis of CaDIRs

Information about the chromosomal location of CaDIRs was obtained from the Pepper Genome Platform (PGP) (http://peppergenome.snu.ac.kr/) as described by Zhang *et al*.^30^, and the genes were mapped on chromosomes using MapDraw^39^. A duplication analysis within the pepper genome was performed with the criteria depicted by Gu *et al*.^40^: (1) the FASTA-alignable region between the two proteins should be longer than 80% of the longer protein, and (2) the identity between the two proteins (I) should be I ≥ 30% if the alignable region is longer than 150 aa and I ≥ 0.01*n* + 4.8 L^−0.32(1+exp(−L/1000)^ ^41^ if otherwise, where n = 6 and L is the alignable length between the two proteins^40,41^.

### Analysis of Conserved Motifs of CaDIRs

The CaDIR-conserved domains were confirmed by Pfam (http://pfam.xfam.org/) and SMART (http://smart.embl-heidelberg.de/). The identification of the conserved motifs was determined as described by Guo *et al*.^35^ with the maximum number of motifs = 10.

### Search for cis-acting Elements in the Promoters of CaDIRs

As described by Guo *et al*.^35^, 1500 bp upstream regions from the start codon (ATG) of the CaDIRs were derived from PGD and used as a query to search the Cis-acting regulatory elements. Cis-element analysis of CaDIRs was conducted using the online program PlantCARE (http://bioinformatics.psb.ugent.be/webtools/plantcare/html/)^42^.

### Plant Materials and Seedlings Treatment

The plant materials, *P*. *capsici* strains and inoculation method used in the current research work are the same as those described by Zhang *et al*.^30^. Root samples were collected at 0, 6, 12, 24 and 48 hours post inoculation (hpi) for RNA extraction. For the plant-signaling molecule treatments, 5 mM of salicylic acid (SA) or 50 μM of methyl jasmonate (MeJA) solutions and sterile ddH_2_O were sprayed on the pepper plants. The leaf samples for RNA extraction were collected at different time points (0, 3, 6, 9, 12, 24 and 48 hours post treatment (hpt)). For drought and salt stress, the roots of seedlings were watered with 300 mM each of mannitol and NaCl solutions, respectively, while sterile water was used in the control plants, and the root samples from the treated and control plants were collected at various times (0, 3, 6, 9, 12, 24 and 48 hpt) for RNA extraction. For tissue-specific expression, samples were collected from leaves, stems, flowers, roots, green fruits and red fruits. All of the collected samples were immediately frozen in liquid nitrogen and stored at −80 °C for RNA extraction. All treatments were performed and analyzed thrice in separate experiments.

### *P*. *capsici* Preparation and Inoculation

*P*. *capsici* was prepared as described by Zhang *et al*.^43^. The detached leaf inoculation assay was conducted as described in our previous study^44^ and stored in a growth chamber at 28 °C with 60% relative humidity and a 16 h light/8 h dark photoperiod.

### RNA Extraction and qRT-PCR analysis

Total RNA was extracted using the method of Guo *et al*.^45^, and the synthesis of cDNA was conducted according to the instructions of the manufacturer’s Prime Script Kit (Takara, Dalian, China). To check the quality and concentration of the cDNA, the Nano-Drop (Thermo Scientific Nano-Drop 2000C, USA) instrument was used. For the real-time quantitative PCR (qRT-PCR) analysis, Primer Premier 5.0 software was used to design the primer pairs (Supplementary Table S2) for CaDIRs, and their specificities was evaluated online using NCBI Primer BLAST (https://www.ncbi.nlm.nih.gov/tools/primer-blast/). The internal control used in the study was a pepper ubiquitin-conjugating protein gene (*CaUbi3*)^46^. The qRT-PCR was performed as described by Guo *et al*.^31^ with a slight modification of the annealing temperature (59 °C for 30 s). The relative expression levels of all of the CaDIRs were calculated using the 2^−ΔΔCT^ method^47^.

### Measurement of the acid-soluble lignin content after *P*. *capsici* inoculation

The samples (whole plants) were collected at different times (0, 6, 12, 24, 48, and 72 h) with *P*. *capsici* post-inoculation (pi) and oven dried at 80 °C for 24 h. The acid-soluble lignin content was measured by the Klason method as described by Wu *et al*.^28^.

### VIGS Assay of *CaDIR7* in Pepper

The VIGS system was used for the knock-down of the *CaDIR7* gene in the pepper plant cultivar AA3, and the VIGS assay was performed as described by Liu *et al*.^48^. Briefly, the 3′-untranslated region (UTR) of the *CaDIR7* gene was cloned into a *pTRV2* vector to construct the recombinant plasmid *pTRV2:CaDIR7*, which was further used in the subsequent research to ensure the specific silencing of *CaDIR7* (primer pairs used for vector construction are shown in Supplementary Table S3). Afterwards, the freeze-thaw method was used to transform *pTRV1*, *pTRV2* (negative control), and *pTRV2:CaPDS* (positive control) along with the combined vector *pTRV2:CaDIR7* into an *Agrobacterium tumefaciens* strain (GV3101). *A*. *tumefaciens* strain (GV3101) harboring *pTRV1* was mixed at a 1:1 ratio with *pTRV2*, *pTRV2-CaPDS* and *pTRV2-CaDIR7*. The *agrobacterium* inocula suspensions harboring *pTRV1*, *pTRV2:00*, *pTRV2:CaPDS* or *pTRV2:CaDIR7* (OD600 = 1.0) were infiltrated into the cotyledons of pepper plants using a 1.0 mL sterilized needleless syringe. Then, these infiltrated pepper plants were grown in a growth chamber under the same growing conditions as described by Wang *et al*.^49^. Forty-five days post-infiltration, leaf samples from the control and *CaDIR7*-silenced plants were collected to measure the silencing efficiency. The experiment was conducted with three biological replicates.

### Determination of Root Activity

The triphenyltetrazolium chloride (TTC) method was used to measure the root activity^50^. Before the TTC test, root tips (approximately 0.2 g) from the control (TRV:00) and *CaDIR7*-silenced (*pTRV2*:*CaDIR7*) plants were collected at various times after the inoculation with the *P*. *capsici* avirulent strain (PC), washed with ddH_2_O and then gently dried with moisture-absorbent paper. Afterwards, a modified TTC method was used to measure the root activity as described by Jin *et al*.^44^. The treatments were conducted in three biological replications, and measurements were repeated thrice.

### Measurement of relative electrolyte leakage (REL) and chlorophyll content

The relative electrolyte leakage (REL) was measured according to the method described by Yin *et al*.^51^. REL was computed as follow: REL (%) = C_1_/C_2_ × 100. To measure the chlorophyll content, leaf disks (0.5 cm) from the control and *CaDIR7*-silenced plants were floated in different concentrations (0, 100, 300 mM) of NaCl and Mannitol solutions at 25 °C for 72 hours. The total chlorophyll contents of the samples (0.2 g) were spectrophotometrically measured after extracting into 80% (v/v) acetone as described by Guo *et al*.^52^.

### Statistical analysis

The data were subjected to an analysis of variance (ANOVA) using SPSS software (SPSS version 16.0, SPSS Inc., U.S.A.). The analyzed data were expressed as the means ± standard deviation (SD) of three replicates in all measured parameters. A least significant difference (P < 0.05) test was used to identify significant differences among the treatments.

## Results

### Identification and Annotation of Dirigent Genes in Pepper

The Hidden Markov Model (HMM) profile of the dirigent domain (Accession no. PF03018) was blast-searched in the pepper genome to identify the dirigent gene family members in pepper. As a result, 26 and 25 dirigent/dirigent-like genes were found in the CM334 and Zunla-1 databases, respectively. Next, these gene sequences were aligned to avoid repetition and alternative splicing. In the case of Zunla-1, out of 25 genes, the single gene (Capana01g000149) sequence exhibited poor identification with CM334 (CA01g01690). When this was used as a query to blast in NCBI Conserved Domain Database (CDD), its domain (Uncharacterized protein family UPF0016) was found to be other than dirigent. Subsequently, primer pairs **(**Supplementary Table S1) were designed to amplify and confirm the doubtful gene sequences through cloning and sequencing. Finally, all of the predicted and sequenced genes protein sequences were confirmed through a blast search in NCBI CDD (https://www.ncbi.nlm.nih.gov/Structure/cdd/wrpsb.cgi). In conclusion, 24 CaDIRs were confirmed in pepper and assigned names on the basis of their chromosomal position and order (Table 1).

**Table 1:** List of 24 CaDIR and CaDIR-like genes identified in pepper and their sequence characteristics.

No.	Name	Gene ID	Chr	ORF	AA	MW	pI	Instability index	Introns	N-Glyc (Asn) position	Localization predicted
1	*CaDIR1*	CA01g01700	1	465	154	17.50	5.62	27.94	0	17, 30, 105	cyto
2	*CaDIR2*	CA01g01710	1	570	189	21.23	7.68	27.65	0	55, 68, 143	chlo
3	*CaDIR3*	CA01g01720	1	558	185	20.88	6.55	23.96	0	48, 56, 61, 121	chlo
4	*CaDIR4*	CA01g15980	1	522	173	19.07	8.78	12.81	0	5	chlo
5	*CaDIR5*	CA01g16630	1	573	190	21.12	9.30	29.24	0	130	extr
6	*CaDIR6*	CA02g19860	2	579	192	20.95	9.25	24.22	0	53	vacu
7	*CaDIR7*	CA03g02390	3	750	249	26.09	5.46	33.6	1	46	nucl
8	*CaDIR8*	Capana04g001233★	4	573	190	21.32	8.76	24.49	0	59, 69, 130, 174	chlo
9	*CaDIR9*	CA05g07510	5	561	186	20.50	8.71	37.16	0	31, 79, 179	chlo
10	*CaDIR10*	CA05g18980	5	687	228	25.14	6.83	43.93	0	24, 35, 62, 92	chlo
11	*CaDIR11*	CA05g18990	5	675	224	23.90	5.22	30.92	0	—	nucl
12	*CaDIR12*	Capana06g000465★	6	993	330	33.04	4.66	24.92	1	120	chlo
13	*CaDIR13*	CA06g10690	6	576	191	20.99	6.03	27.77	0	94, 131	cyto, extr
14	*CaDIR14*	CA07g11710	7	531	176	19.39	5.52	28.73	0	31, 44, 116, 170	extr
15	*CaDIR15*	Capana08g002690★	8	552	183	18.67	8.00	12.29	0	51, 65, 85, 132, 176	cyto, extr
16	*CaDIR16*	CA08g18950	8	582	193	20.71	6.72	17.47	0	62, 72, 186	vacu
17	*CaDIR17*	CA08g18960	8	321	106	11.58	5.16	24.3	0	48, 91	extr
18	*CaDIR18*	CA09g04150	9	567	188	20.83	6.36	36.71	0	57, 67, 128, 172	extr
19	*CaDIR19*	CA09g06650	9	489	162	17.78	5.94	33.41	1	—	cyto
20	*CaDIR20*	CA10g05650	10	579	192	21.20	9.28	18.19	0	59, 95, 130	extr
21	*CaDIR21*	CA11g13500	11	690	228	22.73	4.76	23.76	1	—	cyto
22	*CaDIR22*	CA11g18860	11	450	149	16.40	8.63	28.21	0	23,31,89	cyto
23	*CaDIR23*	Capana12g000353★	12	759	252	26.46	6.17	27.17	1	20, 53	chlo, extr
24	*CaDIR24*	CA12g07560	12	585	194	20.85	9.07	27.49	0	—	chlo

Chr: chromosome; ORF: open reading frame; AA: amino acid; MW: molecular weight (kDa); *p*I: isoelectric point. Sequenced IDs marked with Pentagram (★) are from Zunla-1 genome and others are from CM334 genome.

### Bioinformatics analysis, exon/ intron structure determination and phylogenetic tree construction

**Figure 1:**
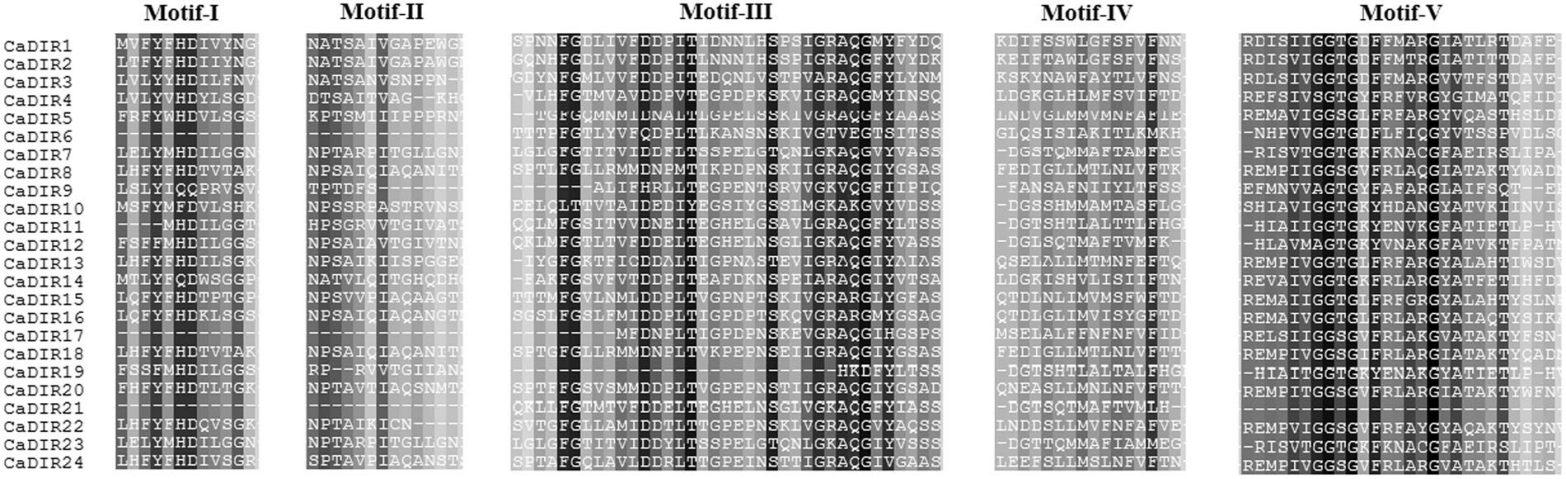
Five conserved characteristic motifs (I-V) of dirigent proteins in pepper CaDIR protein sequences.

**Figure 2:**
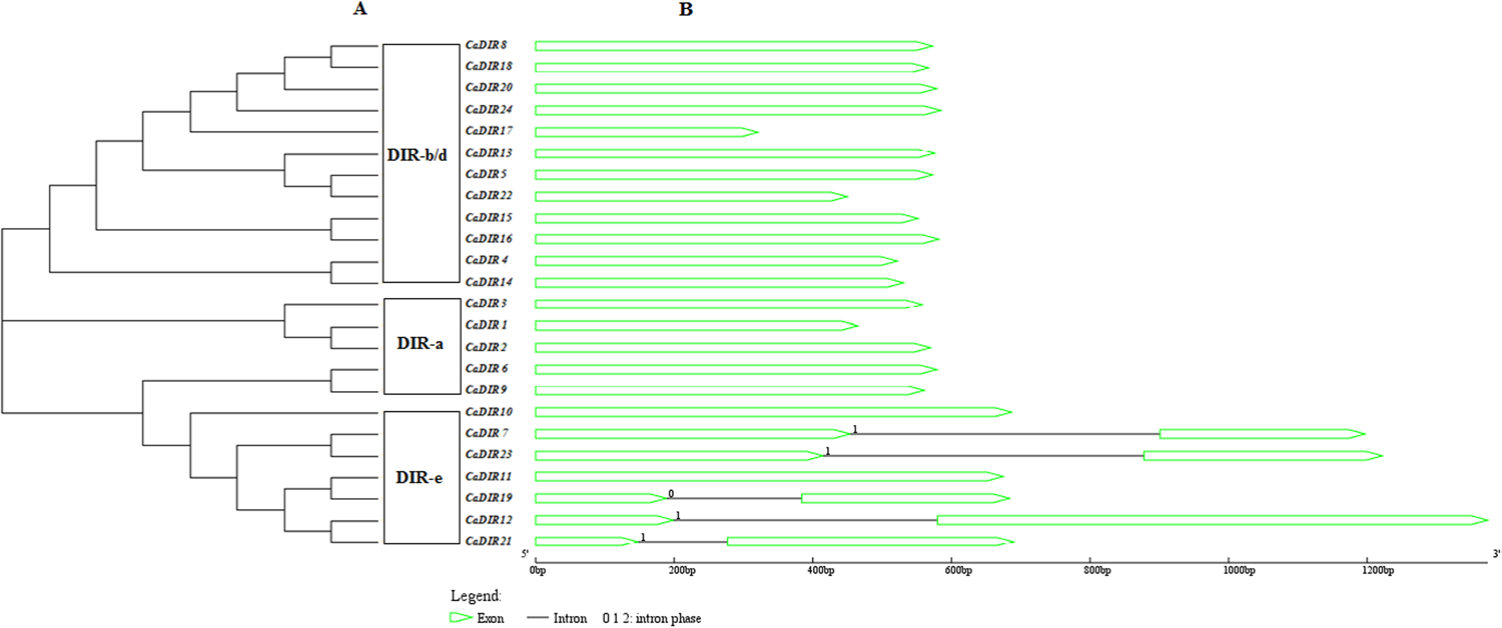
Phylogenetic tree and exon-intron analysis of pepper CaDIRs (**A**) Phylogenetic tree (**B**) exon-intron analysis.

Five well-conserved motifs were found in the amino acid sequences of 24 CaDIRs (Fig. 1). The CDS of CaDIRs ranged from 321 bp (*CaDIR17*) to 993 bp (*CaDIR12*), whereas the deduced proteins had 106 to 330 amino acids. The predicted *p*I values ranged from 4.66 to 9.30, MW ranged from 11.58 to 33.04 kDa and the instability index varied from 12.29 to 43.93 (Table 1). Most of the CaDIRs (except *CaDIR11*, *19*, *21* and *24*) contained Asn sites, indicating that CaDIRs are probably secreted proteins. We anticipated all 24 CaDIRs through the special pathway for extracellular release or their probable final localization in the chloroplast, extra cellular region, nucleus, and cytoplasmic and vacuolar locations (Table 1). The molecular formula and total number of items of the deduced protein are shown in (Supplementary Table S4). The SMART results show that transmembrane regions existed in *CaDIR2*, *3*, *4*, *9*, *10*, *12*, *13*, *14*, *16*, *23* and *24*. The molecular formula showed that *CaDIR3* contained the highest (11) sulfur elements while *CaDIR11* and *19* had only two sulfur elements each. To elucidate the topological structures and evolutionary relationships of the CaDIRs, multiple sequence alignments of amino acid sequences were used to build a Neighbor-Joining (NJ) tree (Fig. 2A). The exon/intron structure showed that out of 24 CaDIRs, 19 genes (79.17%) had no introns while five (20.83%) genes contained only one intron (Fig. 2B). Interestingly, all of the intron containing CaDIRs belonged to the DIR-e group.

**Figure 3:**
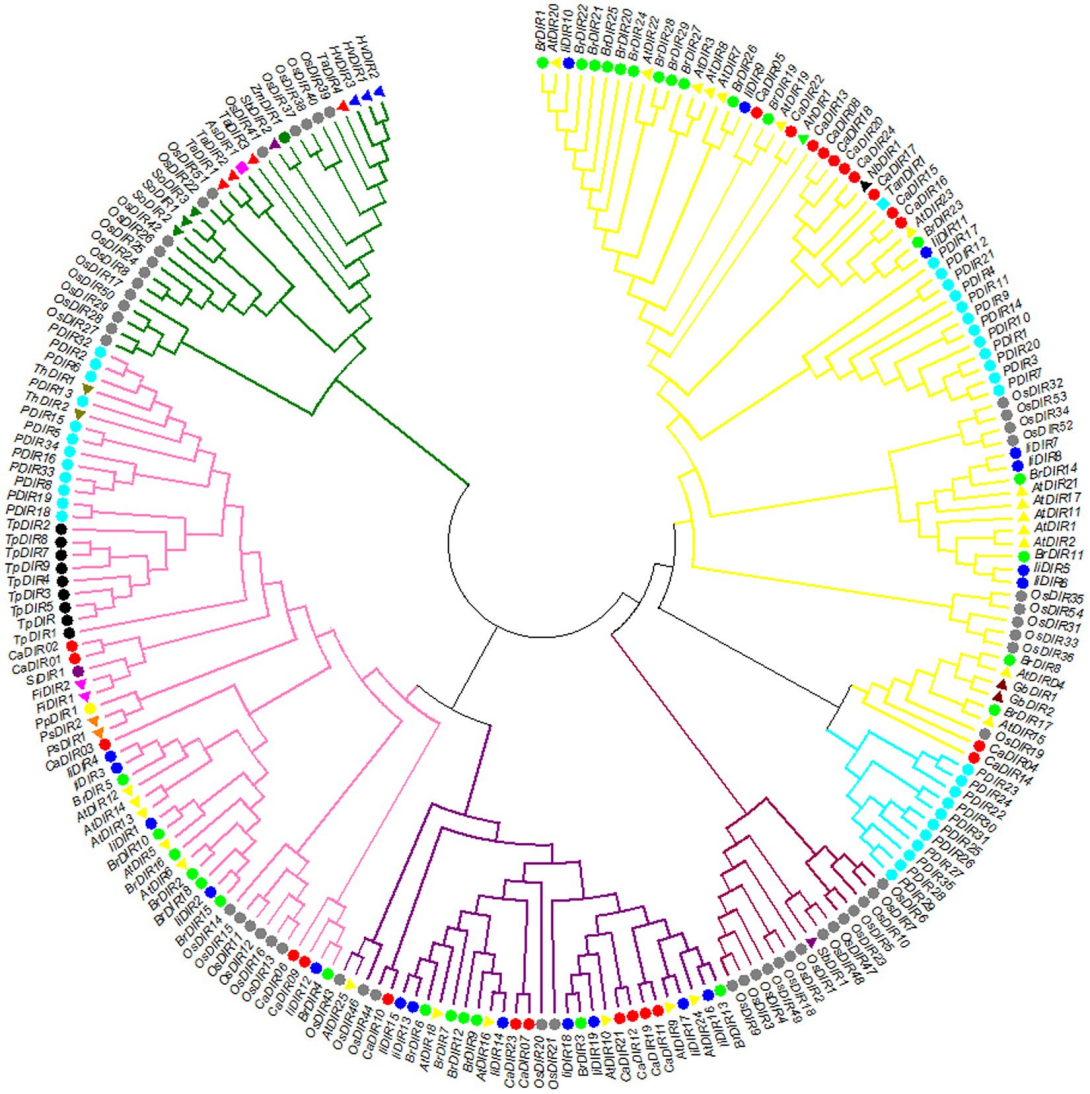
Phylogenetic tree of DIR and DIR-like genes from pepper and other plant species. Protein sequences of 220 dirigent or dirigent-like (DIR) proteins were analyzed by the Maximum Likelihood (ML) test using MEGA 6.0. The subfamilies DIR-a, DIR-b/d, DIR-c, DIR-e, DIR-f and DIR-g are indicated by light pink, yellow, green, purple, sky cyanide and dark pink branch lines, respectively. The nomenclature of the DIRs used in the tree is as follows: Ah, *Arachis hypogaea*; As, *Agrostis stolonifera*; At, *Arabidopsis thaliana*; Br, *Brassica rapa*; Ca, *Capsicum annuum*; Fi, *Forsythia intermedia*; Gb, *Gossypium barbadense*; Hv, *Hordeum vulgare*; Ii, *Isatis indigotica*; Nb, *Nicotiana benthamiana*; Os, *Oryza sativa*; P, *Picea glauca*, *Picea sitchensis* or *P*. *glauca x engelmannii*; Pp, *Podophyllum peltatum*; Ps, *Pisum sativum*; Sb, *Sorghum bicolor*; Si, *Sesamum indicum*; So, *Saccharum officinarum*; Ta, *Triticum aestivum*; Tan, *Tamarix androssowii*; Th, *Tsuga heterophylla*; Tp, *Thuja plicata* and Zm, *Zea mays*. Different species are labeled with different color display markers while CaDIRs are labeled with red circle display markers.

To better understand the similarities and differences in CaDIRs between pepper and other plants, an unrooted phylogenetic tree was generated using 220 DIR and DIR-like gene protein sequences from various plant species. These sequences were from *Arabidopsis thaliana*, *Agrostis stolonifera*, *Arachis hypogaea*, *Forsythia intermedia*, *Picea glauca*, *Hordeum vulgare*, *Nicotiana benthamiana*, *Gossypium barbadense*, *Brassica rapa*, *Isatis indigotica*, *Oryza sativa*, *Pisum sativum*, *Podophyllum peltatum*, *Saccharum officinarum*, *Sesamum indicum*, *Sorghum bicolor*, *Tamarix androssowii*, *Thuja plicata*, *Triticum aestivum*, *Tsuga heterophylla* and *Zea mays* (Supplementary Table S5). The analysis showed that 24 CaDIRs were clearly separated into three distinct groups (Fig. 3). Five CaDIRs (*CaDIR1*, *2*, *3*, *6* and *9*) were clustered into subfamily DIR-a with five *A*. *thaliana* and five *I*. *indigotica* genes. Previously, *IiDIR12* was assigned to DIR-e, but in the current study it was close to *BrDIR4* which lies in the DIR-a group; thus, *IiDIR12* was designated a DIR-a. Another seven CaDIRs (*CaDIR7*, *10*, *11*, *12*, *19*, *21* and *23*) were clustered into the subfamily DIR-e with six *A*. *thaliana* and seven *I*. *indigotica* genes. The other 12 CaDIRs (*CaDIR4*, *5*, *8*, *13*, *14*, *15*, *16*, *17*, *18*, *20*, *22* and *24*) were clustered into another subfamily DIR-b/d with fourteen *A*. *thaliana* and seven *I*. *indigotica* genes. The subfamily branches were colored following the previous studies^27,29^. The 24 CaDIRs were classified into three clusters based on sequence relatedness, which suggested that the two methods were in good agreement.

### Tertiary structures and homologs of CaDIRs

**Figure 4:**
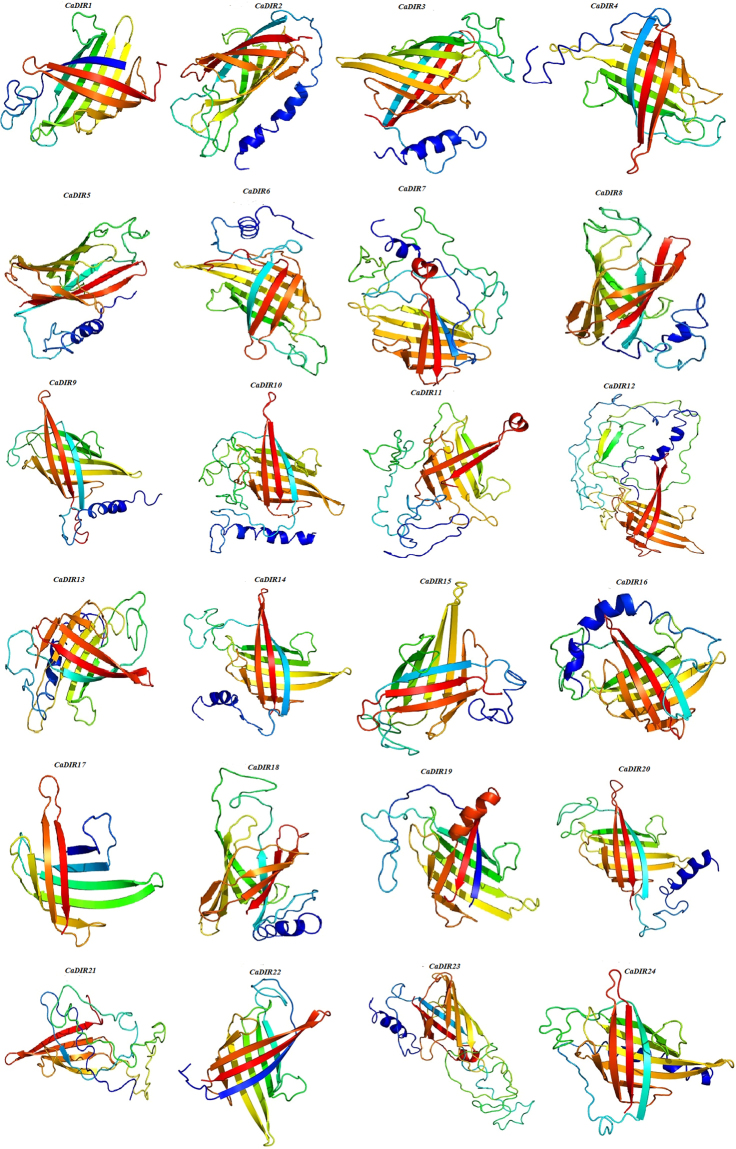
Predicted tertiary structures of pepper CaDIR proteins.

The predicted three-dimensional structures of 24 CaDIR proteins are shown in Fig. 4. Four identical top-scoring proteins for all 24 inquired sequences were found. The hypothetical protein c4revB belonged to disease resistance response protein 206 (drr206) while the other three belonged to the allene oxide cyclase-like protein (AOC) family. Disease resistance response protein 206 (c4revB) shared 20–68% sequence identity with CaDIRs, which was anticipated as a homolog of DIR with 100% probability except *CaDIR21* (99.9%). On the other hand, AOC barrel-like protein d2brja1 shared only 15–27% sequence identity with CaDIRs, and its probability as a DIR homolog was approximately 98.3%, while the probabilities for the other two proteins d1zvca1 and c4h69A were 98.2% and 98.1%, respectively (Table 2).

**Table 2:** The probability and identity of homologous relationship of CaDIRs.

	c4revB (drr206)		d2brja1 (AOC)		c4h69A (AOC)		d1zvca1 (AOC)	
	% confidence	% identity	% confidence	% identity	% confidence	% identity	% confidence	% identity
*CaDIR1*	100.0	33	97.9	21	97.9	22	97.8	21
*CaDIR2*	100.0	33	97.9	24	97.9	18	97.8	26
*CaDIR3*	100.0	31	97.7	18	97.7	22	97.7	20
*CaDIR4*	100.0	33	97.8	19	97.7	24	97.6	21
*CaDIR5*	100.0	30	98.3	22	98.0	20	98.2	27
*CaDIR6*	100.0	29	97.8	23	97.7	20	97.7	20
*CaDIR7*	100.0	34	97.9	21	97.8	18	97.8	18
*CaDIR8*	100.0	32	97.7	24	97.7	22	97.7	25
*CaDIR9*	100.0	68	97.7	15	97.7	25	97.6	19
*CaDIR10*	100.0	60	97.7	22	97.7	23	97.7	22
*CaDIR11*	100.0	53	97.8	22	97.7	20	97.7	20
*CaDIR12*	100.0	31	98.1	22	97.9	22	98.1	24
*CaDIR13*	100.0	31	98.0	19	97.9	23	97.9	18
*CaDIR14*	100.0	36	98.0	26	98.0	17	98.0	20
*CaDIR15*	100.0	35	98.0	26	97.8	21	97.9	22
*CaDIR16*	100.0	33	98.0	24	97.9	22	98.0	24
*CaDIR17*	100.0	30	97.7	27	97.5	28	97.3	27
*CaDIR18*	100.0	40	98.0	20	97.8	21	98.0	20
*CaDIR19*	100.0	23	98.3	19	98.1	20	98.2	22
*CaDIR20*	100.0	29	97.9	21	97.9	20	97.9	21
*CaDIR21*	99.9	28						
*CaDIR22*	100.0	24	97.6	24	97.3	16	97.3	21
*CaDIR23*	100.0	23	97.6	18	97.4	19	97.4	18
*CaDIR24*	100.0	20	97.8	23	97.4	23	97.4	23

The c4revB are disease resistance response proteins 206 (drr206), and d2brja1, c4h69A and d1zvca1 are allene oxide cyclase-like proteins (AOC barrel-like).

### Chromosomal Location and Gene Duplication of CaDIRs

**Figure 5:**
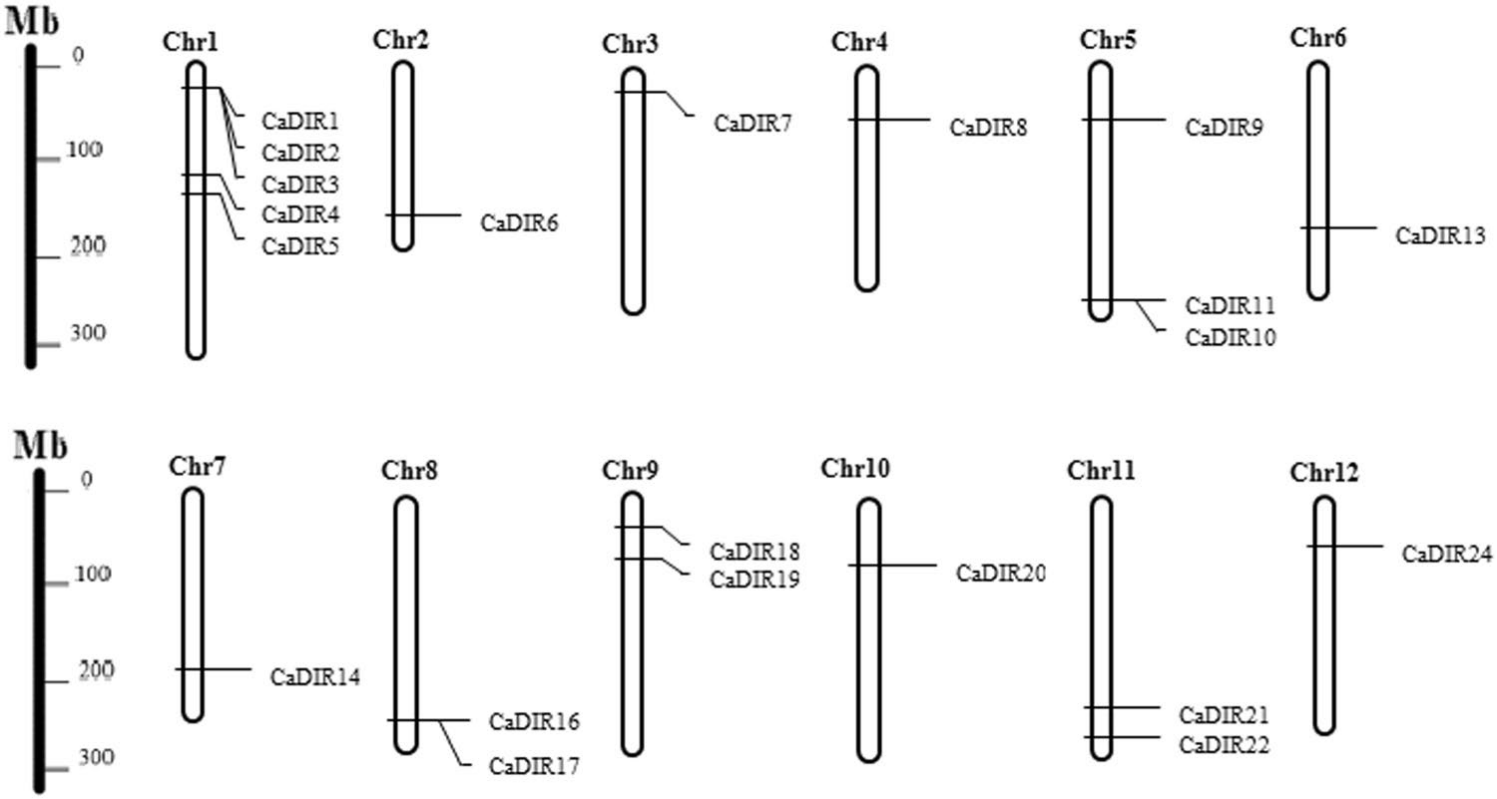
Distribution of CaDIRs on twelve chromosomes of pepper. Three genes (*CaDIR12*, *15* and *23*) were from the Zunla-1 database and located on chromosome 6, 8 and 12, respectively, so they were not mentioned in the figure. The scale represents mega bases (Mb), and the chromosome numbers are indicated at the top of each bar.

According to the chromosomal location of dirigent genes in pepper, the 24 CaDIRs were distributed across all 12 chromosomes of pepper (Fig. 5). The results showed that chromosome 1 had the highest number of genes (20.83%) compared with the other chromosomes. There were three genes (12.5%) on chromosome 5, while two genes each were found on chromosome 8, 9 and 11. The remaining chromosomes (2, 3, 4, 6, 7, 10 and 12) each contained one gene. Three genes (*CaDIR12*, *15* and *23*) located on chromosome 6, 8 and 12, respectively, were from the Zunla-1 database, so they were not mentioned in the figure.

The duplication analysis in Table 3 shows that *CaDIR5* had more segmental duplication events than other genes, and they occurred on chromosome 6, 8, 9 and 10. *CaDIR4*, *14* and *16* each had three segmental duplications. Another three genes (*CaDIR7*, *13* and *18*) had undergone two segmental duplications, respectively. Meanwhile, *CaDIR11* and *20* had one segmental duplication event each. There were 2 clusters of tandemly duplicated CaDIRs, which occurred on chromosome 1. *CaDIR15* also had one segmental duplication event (not shown in the figure). Taken together, our findings suggest that in the expansion of pepper CaDIRs, tandem and segmental duplication have an important contribution.

**Table 3:** Paralogous groups of CaDIRs in pepper.

	Paralogous Group	Gene name
Segmentally duplicated	1	*CaDIR4*, *CaDIR13*, *CaDIR14*, *CaDIR18*
	2	*CaDIR5*, *CaDIR13*, *CaDIR16*, *CaDIR18*, *CaDIR20*
	3	*CaDIR7*, *CaDIR11*
	4	*CaDIR8*, *CaDIR15*
	5	*CaDIR11*, *CaDIR21*
	6	*CaDIR13*, *CaDIR2*, *CaDIR18*
	7	*CaDIR14*, *CaDIR13*, *CaDIR16*, *CaDIR3*
	8	*CaDIR16*, *CaDIR18*, *CaDIR20*, *CaDIR24*
	9	*CaDIR18*, *CaDIR20*, *CaDIR24*
	10	*CaDIR20*, *CaDIR24*
Tandemly duplicated	1	*CaDIR1*, *CaDIR2*, *CaDIR3*
	2	*CaDIR4*, *CaDIR2*

Paralogous groups of segmentally and tandemly duplicated CaDIRs in pepper.

### Identification of conserved motifs of CaDIRs in pepper

**Figure 6:**
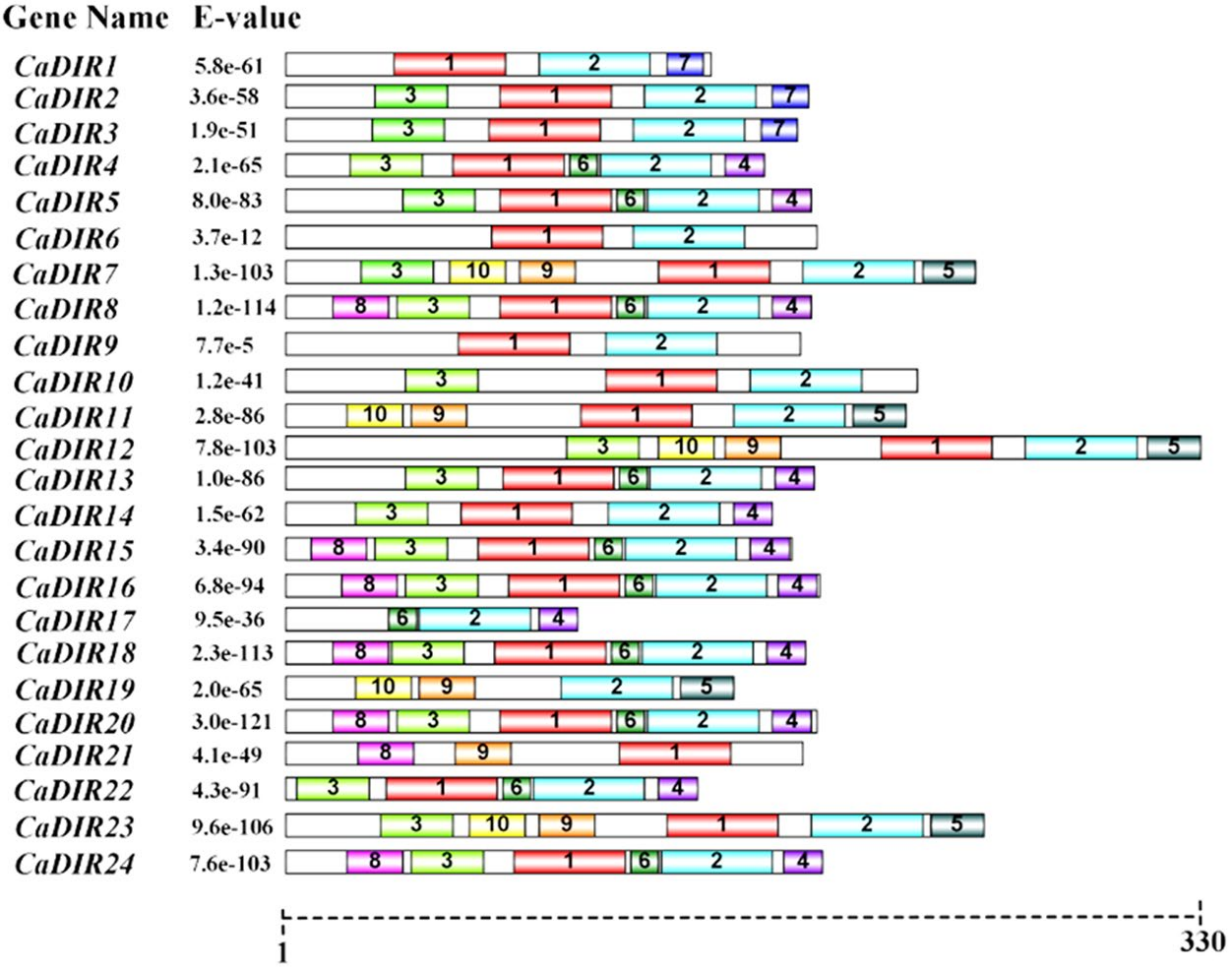
Distribution of conserved motifs of pepper CaDIR members. Each of the ten putative motifs is represented by a number in the colored box. The names of all CaDIRs along with their combined E-values are shown at the left side of the figure, and the motif size scale is at the bottom of the figure. For more details on the motifs refer to Table 5 and Supplementary Table S5.

The conserved motifs of CaDIR proteins were found through the online MEME server (http://meme-suite.org/tools/meme). Ten distinct motifs were identified (Fig. 6, Table 4). The details of the putative motifs are shown in Supplementary Table S6. Motif1 (except *CaDIR17* and *19*) and motif2 (except *CaDIR21*) were found in nearly all of the CaDIRs. Motif3 was also present in most of the CaDIRs except *CaDIR1*, *6*, *9*, *11*, *17*, *19* and *21*. Motif4 and 6 were present in 11 CaDIRs. Motif5 and motif10 were each found in 5 CaDIRs. Motif8 and motif9 existed in 7 and 6 CaDIRs, respectively, while motif7 only existed in 3 CaDIRs. *CaDIR6* and *9* contained the lowest number of motifs (2), and they were motif1 and motif2.

**Table 4:** Motif sequences identified by the MEME Suite.

Motif	Length (aa)	Sequence
1	41	FGTLTMCDDPLTEGPEPNSKIIGRAQGMYVYSSQDDLSQWM
2	41	GSSLSFFGCNPIMHKYREMPIIGGTGKFRMARGYATAKTYW
3	27	KQKMTKLHFYWHDWLSGKNPSAIPICQ
4	15	TGDAIVEYNVVVLHY
5	20	DQHTTDGVETILHITVYLTY
6	11	MNFVFTEGKYN
7	14	YFRLCVDIKLYECW
8	21	PTTHGWEQEPKGVEKWFKRLP
9	21	NGNNPIVNNNNYPFLTGLSGT
10	21	YSGQVPFAKPNGQQPPKNGGV

Motif numbers corresponded to the motifs in Fig. 6.

### Cis-acting elements in the promoter regions of CaDIRs

To investigate the possible cis-elements involved in the activation of defense-related genes, upstream regions of all CaDIRs were analyzed online with Plant CARE. The silico analysis revealed that CaDIRs contained cis-elements conferring responsiveness to plant hormones and biotic and abiotic stresses. As shown in Tables 5 and 6, the defense and stress responsiveness elements (TC-rich repeats) were found in the promoters of all CaDIRs except *CaDIR4*, *14*, *15* and *17*, in which the TC-rich repeats in the promoter region of *CaDIR3* was highest (6) followed by *CaDIR5* and *9* (each having 5). MeJA-responsiveness elements (TGACG-motif) were found in the promoter region of 19 CaDIRs, where *CaDIR2* and *9* had the highest number (4) of elements followed by *CaDIR7*, *11*, *12*, *15*, *16*, *18* and *21* (each having 3). Cis-acting elements involved in salicylic acid responsiveness (TCA-element) and heat stress responsiveness elements (HSE-element) were found in the promoter regions of 16 CaDIRs. The MYB binding site involved in drought-inducibility (MBS), GA-responsive element (GARE-motif) and fungal elicitor-responsive element (W box) were found in the promotor regions of 15, 13 and 12 CaDIRs, respectively. Abscisic acid responsiveness elements (ABRE), Ethylene-responsive element (ERE) and GA-responsive element (P box) were found in the promoter regions of 11 CaDIRs. The cis-acting element involved in low temperature responsiveness (LTR) was found in the promoter region of 10 CaDIRs. In addition, wound-responsive element (WUN-motif) and auxin-responsive elements (TGA-element and AuxRR-core) were found in some of the CaDIRs promoter regions. All of the predicted cis-elements were involved in the response to signaling hormones and stresses.

**Table 5:** Cis-acting elements in the promoter regions of CaDIR genes.

Gene name	TGACG-motif	GARE-motif	TC-rich repeats	TCA-element	MBS	W box	ERE	HSE	ABRE	TGA-element	AuxRR-core	LTR	WUN-motif	P-box
*CaDIR1*	1	1	1	1	0	0	0	0	0	0	0	0	0	0
*CaDIR2*	4	1	3	0	1	1	0	0	0	0	0	0	0	0
*CaDIR3*	0	1	6	0	2	2	1	3	0	0	0	0	0	1
*CaDIR4*	0	1	0	1	1	0	1	0	1	2	0	0	0	0
*CaDIR5*	1	0	5	3	1	0	0	2	0	0	0	0	0	0
*CaDIR6*	1	1	1	1	1	1	0	1	0	0	1	0	0	0
*CaDIR7*	3	1	2	1	0	3	1	2	0	0	1	0	0	1
*CaDIR8*	1	0	1	2	2	0	1	0	0	0	0	1	0	0
*CaDIR9*	4	0	5	2	0	0	0	0	0	1	0	0	0	1
*CaDIR10*	2	0	2	2	2	2	0	3	1	1	0	1	0	0
*CaDIR11*	3	1	1	3	5	0	0	0	1	1	0	1	0	0
*CaDIR12*	3	1	2	0	4	1	1	1	1	1	1	0	1	3
*CaDIR13*	1	0	1	0	0	0	2	5	1	0	0	1	0	1
*CaDIR14*	1	0	0	0	1	1	0	0	0	0	0	1	0	1
*CaDIR15*	3	0	0	2	0	1	0	0	0	0	1	0	0	1
*CaDIR16*	3	1	1	1	2	0	1	2	1	0	0	1	0	0
*CaDIR17*	0	1	0	0	3	0	1	1	1	0	0	1	0	0
*CaDIR18*	3	0	2	1	0	3	4	2	0	1	0	1	0	0
*CaDIR19*	0	2	2	2	1	0	0	3	1	0	0	1	1	1
*CaDIR20*	1	1	1	0	2	1	1	1	0	1	0	0	0	2
*CaDIR21*	3	0	1	1	0	0	0	1	1	0	0	0	0	0
*CaDIR22*	0	1	3	1	0	1	2	1	2	0	0	0	0	0
*CaDIR23*	1	0	2	0	0	1	0	2	1	0	0	0	1	1
*CaDIR24*	1	0	2	1	1	0	0	1	0	1	0	1	0	3

**Table 6:** Function of the cis-elements found in the promoters of CaDIRs.

Cis-acting element	Motif	Function	Reference
Defense-related and stress stimulative elements	TC-rich repeats	Defense and stress responsiveness	^83^
	W box	Fungal elicitor-responsive element	^84^
	WUN-motif	Wound-responsive element	^85^
	HSE	Heat stress responsiveness	^85^
	LTR	Low temperature responsiveness	^86^
	MBS	MYB binding site involved in drought-inducibility	^87^
Plant hormones responsive elements	TGACG-motif	MeJA responsiveness	^75^
	GARE-motif	GA-responsive element	^88^
	TCA-element	Salicylic acid responsiveness	^76^
	TGA-element	Auxin-responsive element	^85^
	ERE	Ethylene-responsive element	^89^
	ABRE	ABA responsiveness	^90^
	AuxRR-core	Auxin responsiveness	^91^
	P-box	GA-responsive element	^92^

### Expression Analysis of CaDIRs under *P*. *capsici*, abiotic and signaling molecules challenges

**Figure 7:**
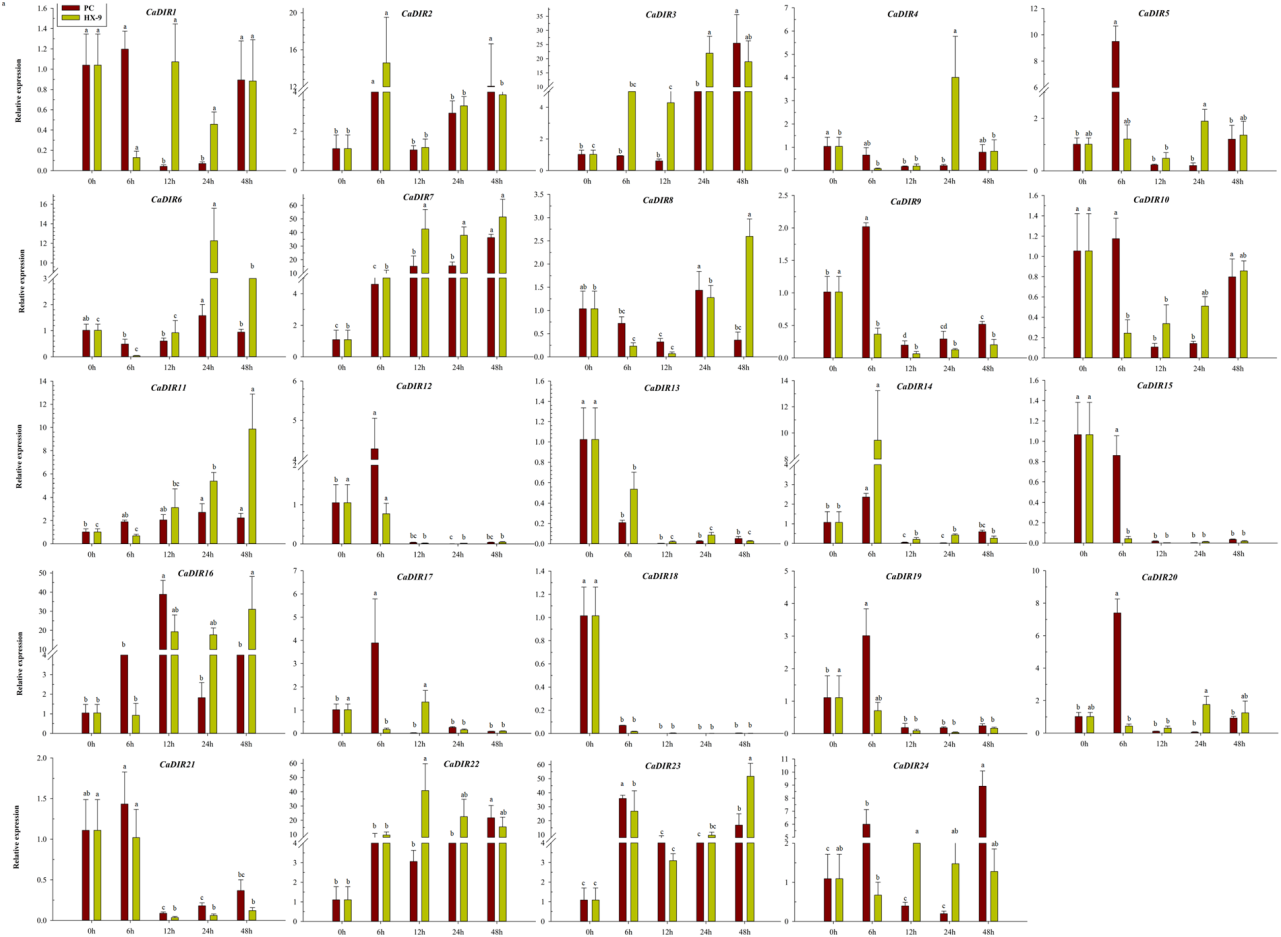
Expression profiles of CaDIRs in response to inoculation with virulent and avirulent *Phytophthora capsici* strains. Mean values and SDs for three replicates are shown. Small letters represent significant differences (p < 0.05).

To explore the effect of *P*. *capsici* infestation on CaDIRs, the pepper cultivar “AA3” was inoculated with virulent and avirulent strains via root drenching, and the expression levels were analyzed by qRT-PCR (Fig. 7). The results revealed that different expression patterns were observed for CaDIRs; therefore, they were categorized into eight groups based on their expression levels. Group-I contained *CaDIR13*, *15* and *18*, which showed complete down-regulation with both strains pi. Another three CaDIRs (*CaDIR1*, *10* and *21*) belonged to Group-II, showed up-regulation in the case of avirulent strain pi at 6 h and then down-regulated. Meanwhile, with the virulent strain pi, they exhibited total down-regulation except *CaDIR1*, which was slightly up-regulated at 12 h and then down-regulated. Group-III (*CaDIR2*, *3*, *22* and *24*) showed up-regulation after avirulent strain pi, in which *CaDIR2*, *22* and *24* were up-regulated at 6 h, followed by down-regulation and then up-regulation to the maximum at 48 h (12.06, 21.69 and 8.92 respectively), while *CaDIR3* was peaked (25.46) at 48 h. In the case of virulent strain pi, *CaDIR22* and *24* were up-regulated, reached a maximum at 12 h (40.89 and 2.71 respectively) and then down-regulated, whereas *CaDIR2* and *3* were up-regulated at 6 h, then showed a downward trend at 12 h, and again up-regulated. Group-IV members comprised *CaDIR8*, *11*, *16* and *23*. The transcription levels of all members of this group were elevated with virulent strain inoculation and reached a maximum at 48 hpi (9.87, 31.02 and 51.71 respectively) except *CaDIR8*, which was initially down-regulated, then up-regulated and finally reached a peak at 48 h (2.60). Meanwhile, in the case of avirulent strain inoculation, *CaDIR8* (1.43) and *11* (2.71) were up-regulated to a maximum at 24 hpi, whereas *CaDIR16* and *23* were up-regulated to a maximum at 12 hpi (38.83) and 6 (35.91) hpi, respectively. Group-V contained six CaDIRs (*CaDIR5*, *9*, *12*, *17*, *19* and *20*), which showed up-regulation at 6 hpi with the avirulent strain and then down-regulated at other time points; meanwhile, with the virulent strain pi, down-regulation was observed for *CaDIR9*, *12* and *19*, whereas *CaDIR17*, *5* and *20* showed slight up-regulation at 12 h and 24 h. Group-VI (*CaDIR4* and *6*) initially exhibited down-regulation after inoculation with the virulent strain, up-regulated to the maximum (4.01 and 12.27 respectively) at 24 h and then down-regulated again, whereas with avirulent pi, both *CaDIRs* showed down-regulation. Group-VII contained *CaDIR14*, which showed up-regulation to a maximum at 6 h for both strains and then down-regulated. Group-VIII contained *CaDIR7*, which exhibited progressive up-regulation for both virulent and avirulent strains and reached a peak (51.43 and 36.28 respectively) at 48 hpi.

**Figure 8:**
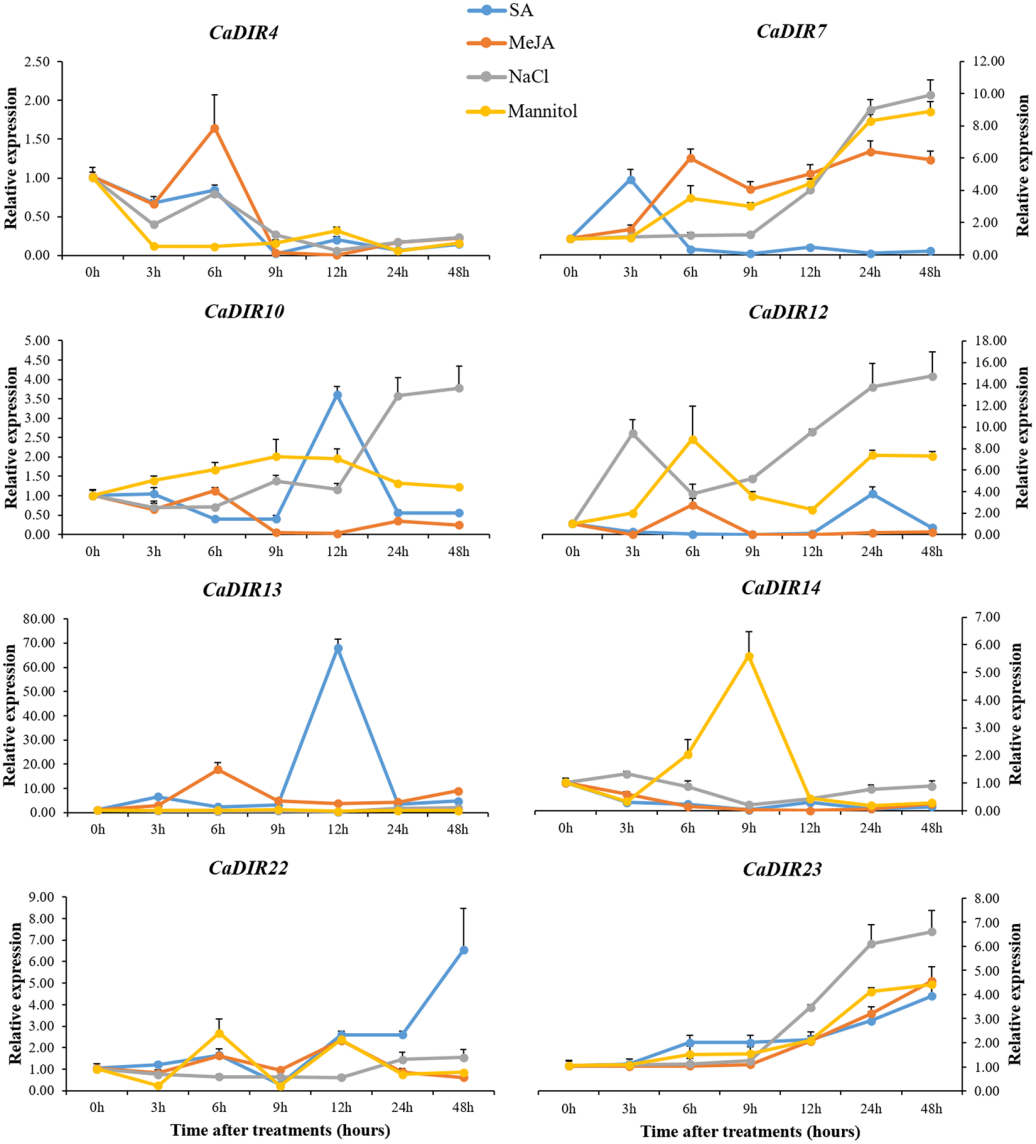
Expression profiles of CaDIRs in response to treatment with SA, MeJA, NaCl and Mannitol. Mean values and SDs for three replicates are shown.

To investigate the effects of abiotic stresses on the expression patterns of CaDIRs, eight representative genes (*CaDIR4*, *CaDIR7*, *CaDIR10*, *CaDIR12*, *CaDIR13*, *CaDIR14*, *CaDIR22* and *CaDIR23*) from each of the eight groups above were subjected to NaCl and mannitol stresses (Fig. 8). The results showed that two candidate CaDIRs (*CaDIR4* and *CaDIR13*) showed no significant response to both stresses, and *CaDIR4* was completely down-regulated. Similarly, *CaDIR14* also showed down-regulation in response to NaCl, whereas it up-regulated at 6 h and 9 h and then down-regulated in response to mannitol. *CaDIR7* and *CaDIR23* were gradually up-regulated by abiotic stresses and reached to peak at 48 hours post treatment (hpt). *CaDIR10* and *CaDIR12* were up-regulated in response to mannitol and were highest at 9 (2.01) hpt and 6 (8.87) hpt, respectively, whereas both peaked (3.77 and 14.73 respectively) at 48 h in response to NaCl. *CaDIR22* was initially down-regulated in response to NaCl but then up-regulated at 24 h and 48 h, whereas in response to mannitol concomitant up- and down-regulation occurred.

The selected CaDIRs from the eight groups above were also exposed to hormonal stress (MeJA and SA). The expression results revealed that in response to signaling molecules, *CaDIR4*, *CaDIR10* and *CaDIR14* showed no significant response, but *CaDIR4* and *CaDIR10* showed slight up-regulation at 6 h and 12 h post MeJA and SA treatments, respectively (Fig. 8**)**. *CaDIR7* and *CaDIR22* showed a significant response to MeJA and SA respectively, reaching a maximum at 24 and 48 hpt, respectively. *CaDIR12* and *CaDIR13* responded to SA and reached a peak at 24 and 12 hpt, respectively, whereas in response to MeJA both peaked at 6 hpt and then showed down-regulation. *CaDIR23* exhibited a progressive increment in the expression levels and reached a maximum (4.56 and 3.94) at 48 hpt.

### Expression analysis of CaDIRs in different tissues of pepper

**Figure 9:**
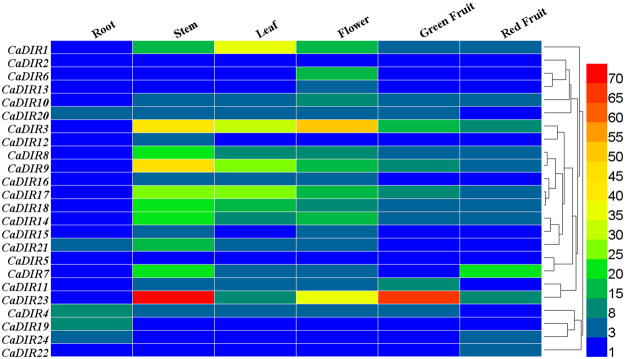
Tissue-specific expression analysis of pepper CaDIRs. The analyzed tissues included root, stem, leaf, flower, green fruit and red fruit.

To investigate the involvement of CaDIRs in the growth and development of pepper, the expression levels in various vegetative and reproductive organs of cultivar AA3 grown under normal conditions were analyzed through qRT-PCR using gene-specific primer pairs (Supplementary Table S2). As shown in Fig. 9, the expression pattern of each CaDIR was different in various organs and stages. The analysis showed that out of 24 CaDIRs, two genes (*CaDIR2* and *5*) were not expressed in all of the tested organs. Five CaDIRs were expressed in one organ only: *CaDIR6* and *13* were expressed in the flower while *CaDIR12*, *19* and *22* were expressed in the stem, root and red fruit, respectively. *CaDIR1*, *3*, *8*, *9*, *10*, *14*, *17*, *18* and *23* were expressed in all of the tested tissues except the root, and *CaDIR4* and* 20* were expressed in all tissues except red fruit. *CaDIR7* was expressed in the leaf, stem, flower and red fruit but showed no expression in the root and green fruit. Similarly, *CaDIR11* was also expressed in the leaf, stem, flower and green fruit. The other CaDIRs were expressed in two or three organs. Among all CaDIRs, *CaDIR*23 was expressed at the highest level in the stem (71.55) and green fruit (66.48). Collectively, CaDIRs showed the highest expression in flowers, followed by the stem, leaf, green fruit and red fruit, whereas they were expressed at the lowest levels in roots.

### Changes in acid-soluble lignin content after *P*. *capsici* inoculation

The acid-soluble lignin content was measured at various times (0, 6, 12, 24, 48 and 72 h) after inoculation with the *P*. *capsici* strains. The results showed that in the case of the avirulent strain, the acid-soluble lignin content was increased by 14.46%, 36.09%, 25.67%, 90.40% and 43.93%, whereas with the virulent strain, the increase was 21.68%, 50.57%, 33.07%, 116.02% and 68.10% compared with control plants at 6, 12, 24, 48 and 72 hpi, respectively (Table 7).

**Table 7:** Changes in the acid soluble (Klason) lignin content of the pepper plants after inoculation with PC and HX-9 strains of *Phytophthora capsici* at different time points.

	0 h (Untreated)	6 h	12 h	24 h	48 h	72 h
Mock-inoculated	6.29 ± 0.44	7.02 ± 0.49	4.77 ± 0.33	5.32 ± 0.37	3.52 ± 0.25	5.02 ± 0.35
PC strain inoculated	6.29 ± 0.44	8.04 ± 0.24	6.50 ± 0.13	6.68 ± 0.27	6.70 ± 0.07	7.23 ± 0.36
Increased Percentages	0	14.46%	36.09%	25.67%	90.40%	43.93%
HX-9 strain inoculated	6.29 ± 0.44	8.54 ± 0.34	7.19 ± 0.14	7.08 ± 0.28	7.60 ± 0.38	8.44 ± 0.25
Increase Percentages	0	21.68%	50.57%	33.07%	116.02%	68.10%

Data are the mean value of three replications with standard deviation (mean ± SD for triplicates).

### Reduced tolerance of *CaDIR7*-silenced pepper plants to *P*. *capsici*

**Figure 10:**
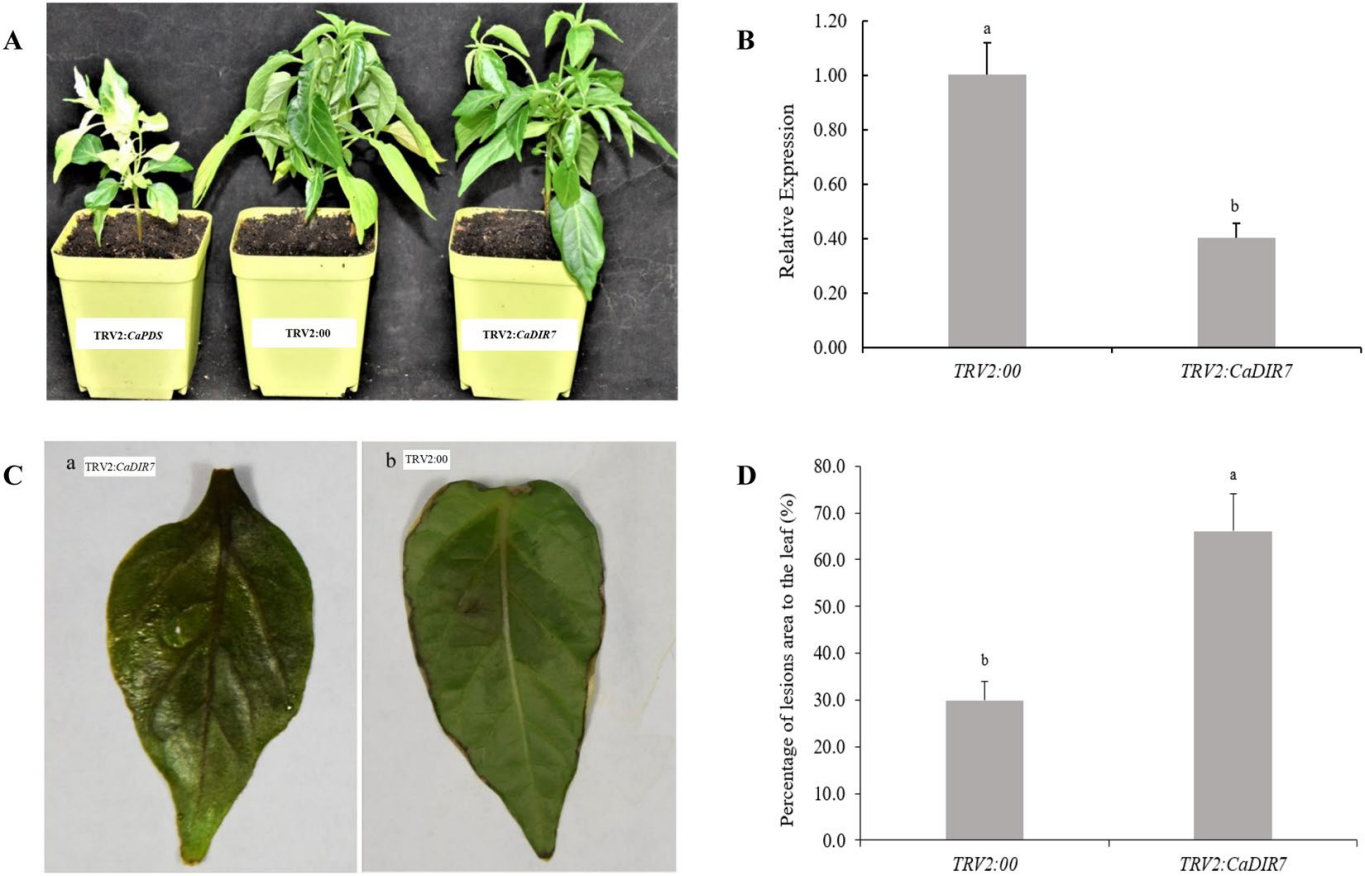
The phenotypes and loss of function analysis of *CaDIR7* in leaves of pepper. (**A**) The phenotypes of TRV2:00, TRV2:*CaPDS* and TRV2:*CaDIR7*, (**B**) The relative expression of *CaDIR7* in leaves of *CaDIR7*-silenced and control (TRV2:00) plants, (**C**) disease symptoms developed on the detached leaves of *CaDIR7*-silenced (a) and control (TRV2:00) (b) plants at 3 days post inoculation (dpi), (**D**) percentage of the lesion area of leaves exposed to *P*. *capsici* infection from TRV2:00 and TRV2:*CaDIR7*. Error bars represent the mean ± SD of three independent biological replicates. Small letters represent significant differences (p < 0.05).

**Figure 11:**
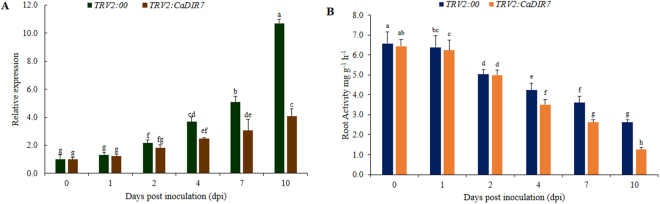
The *CaDIR7*-silenced pepper plants exhibit reduced resistance to *P*. *capsici*. (**A**) The expression level of *CaDIR7* in control (TRV2:00) and *CaDIR7*-silenced plants, (**B**) root activity of the control and *CaDIR7-*silenced plants after inoculation with the avirulent strain of *P*. *capsici*. Values are the means ± SD from three independent experiments. Small letters represent significant differences (p < 0.05).

To determine the loss of function of the *CaDIR7* gene in pepper, the VIGS approach was used for gene knock-down in the pepper cultivar AA3. To confirm the visual success of VIGS, a positive control vector (*pTRV2:CaPDS*) was used for the silencing of the *CaPDS* gene, which produced a typical white color as a result of the photo-bleaching phenotype in the leaves, while the negative control used in the study was *pTRV2:00*. After 45 days of inoculation, the photobleaching phenotypes observed in the leaves of *CaPDS*-silenced plants (Fig. 10A), demonstrating that VIGS was successful. At that point, the silencing efficiency of *CaDIR7* was investigated through a qRT-PCR analysis, and the results showed that the expression levels of *CaDIR7* gene in the silenced plants (*pTRV2*-*CaDIR7*) were much lower than in the control plants (*pTRV-00*) (Fig. 10B). The expression of *CaDIR7* was induced significantly with the interaction of the avirulent strain of *P*. *capsici*. Thus, to ensure the role of *CaDIR7* in defense response, the fourth to fifth leaves from the top of the silenced (*pTRV2*:*CaDIR7*) and control (*pTRV2:00*) pepper plants were removed and infiltrated with 20 µL zoospore suspension (10^4^ zoospores mL^−1^) of the avirulent strain of *P*. *capsici*. Three days post inoculation (dpi), disease lesions were detected on the leaves of both *CaDIR7*-silenced (**a**) and control (**b**) plants, but numerous and larger lesions were found on *CaDIR7*-silenced plants leaves (Fig. 10C). The quantitative analysis of the lesion area showed that detached leaves of the silenced plants exhibited significantly larger lesion area than the control (Fig. 10D). Additionally, the expression level of the *CaDIR7* gene during *P*. *capsici* infection was checked, and the time was expanded to 10 dpi. A significant increase in the expression levels of *CaDIR7* was observed at 2, 4, 7 and 10 dpi in the empty vector control plants versus the *CaDIR7*-silenced plants (Fig. 11A). Moreover, for *P*. *capsici* pi, the root activity of the *CaDIR7*-silenced and empty vector control plants was also measured by the TTC method. The root activity of the silenced plants was lower than the control, and a significant difference was observed at 4, 7 and 10 dpi between the silenced and control plants (Fig. 11B).

### Reduced tolerance of *CaDIR7*-silenced pepper plants to salt

**Figure 12:**
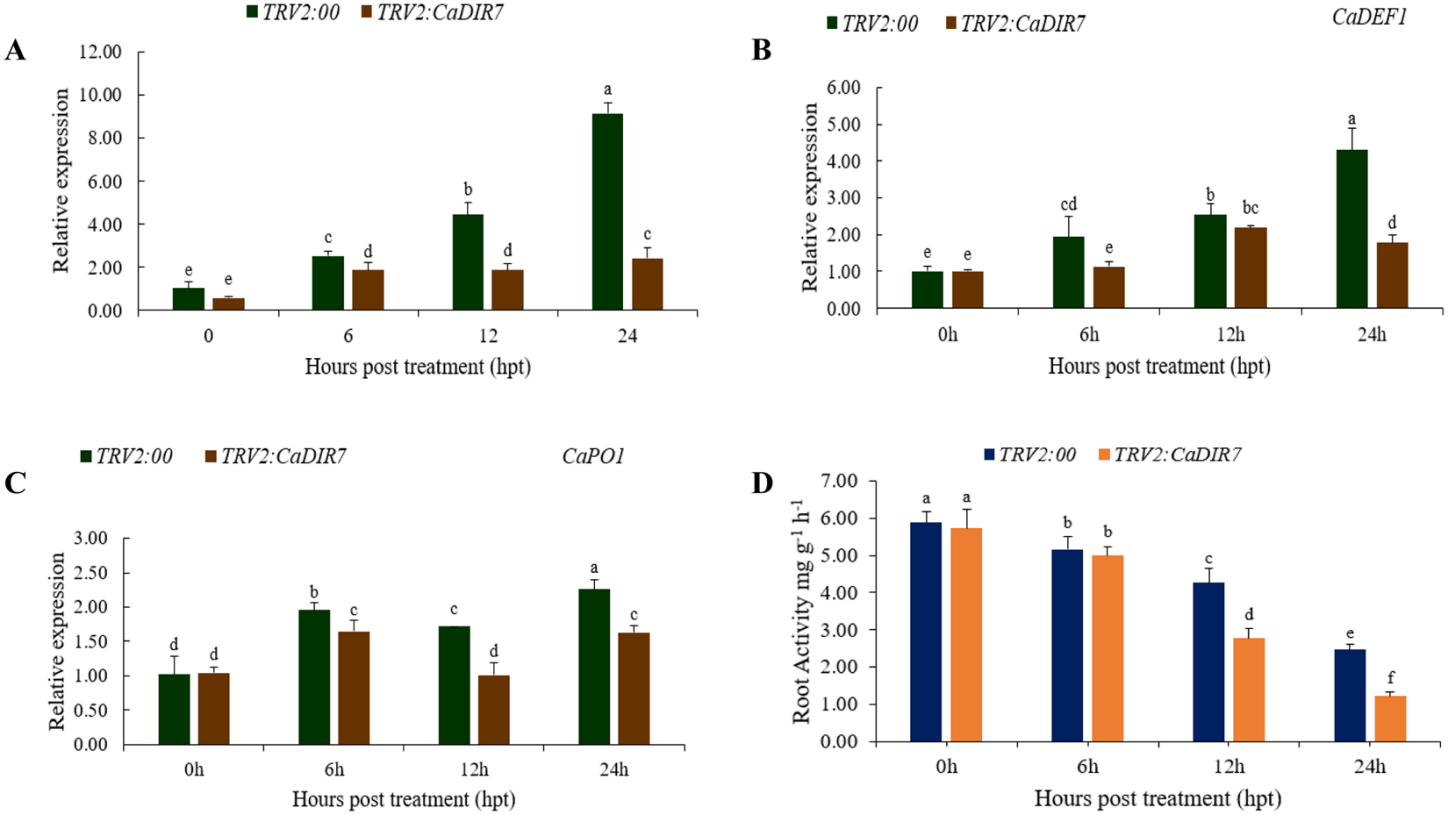
The *CaDIR7-*silenced pepper plants compromised tolerance to NaCl stress. (**A**) The expression level of *CaDIR7*, (**B**) expression level of *CaDEF1*, and (**C**) expression level of *CaPO1* genes in control (TRV2:00) and *CaDIR7*-silenced plants, (**D**) root activity of the control and *CaDIR7-*silenced plants. Values are the means ± SD from three independent experiments. Small letters represent significant differences (p < 0.05).

To assess the function of *CaDIR7* under salt stress, the empty vector control and *CaDIR7*-silenced plants were exposed to NaCl stress (300 mM). As shown in Fig. 12A, the loss of function of *CaDIR7* significantly compromised salt stress tolerance. A higher expression of *CaDIR7* was recorded in the empty vector control plants than in the *CaDIR7*-silenced plants. In addition, the expression levels of other defense-related genes were also examined to see whether the silencing of *CaDIR7* alters their expression. It was found that with the passage of time after salt stress, the transcript levels of *CaDEF1* (a JA dependent gene)^53^ and *CaPO1* (a peroxidase gene)^54^ were elevated, but their elevation in the empty vector control plants was higher than in the *CaDIR7*-silenced plants (Fig. 12B,C). Furthermore, root activity was also measured, and the results showed a significant reduction in the root activity after salt stress. At 24 h post NaCl stress, the root activity of the *CaDIR7*-silenced plants (2.46) was less than half of that of the empty vector control (1.22) (Fig. 12D).

### Effect of silencing of *CaDIR7* on REL and chlorophyll content

**Figure 13:**
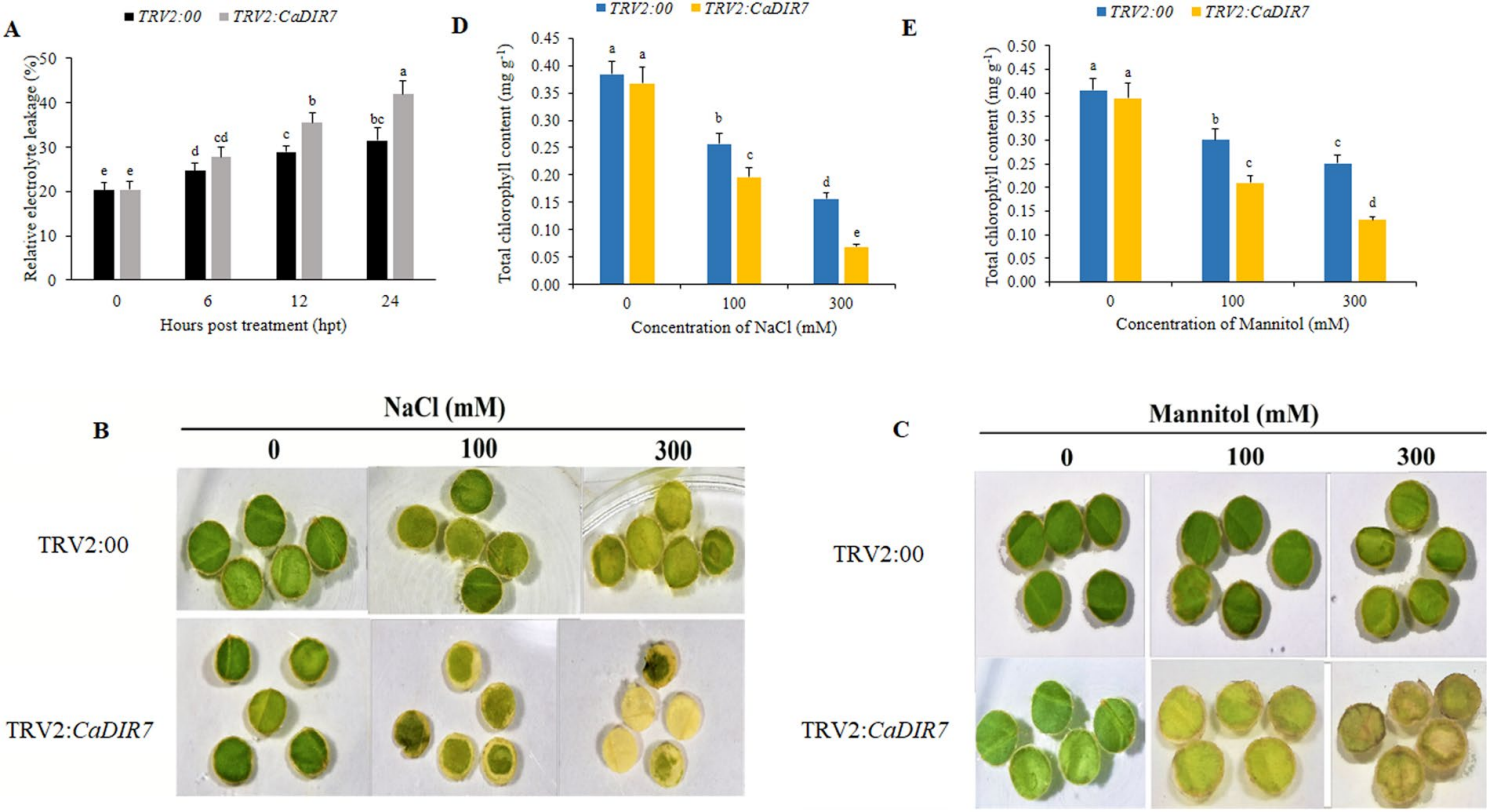
Reduced tolerance of *CaDIR7*-silenced pepper plants to salt and mannitol stresses. (**A**) REL post salt stress of the *CaDIR7-*silenced and control (TRV2:00) plants, (**B**,**C**) leaf discs phenotypes (0.5 cm in diameter), (**D**,**E)** chlorophyll contents of the *CaDIR7*-silenced and control plants in response to 100 mM and 300 mM NaCl and mannitol stresses after 48 h, respectively. Values are the means ± SD from three independent experiments. Small letters represent significant differences (p < 0.05).

The extent of membrane damage caused by NaCl stress was assessed by an indirect measurement of electrolyte leakage. After salt stress, leaf discs were collected from TRV2:00 and TRV2:*CaDIR7* plants at different times for REL measurement. The results showed excessive electrolyte leakage in TRV2:*CaDIR7* versus TRV2:00 plants. At 24 h post salt stress, the REL was significantly higher in the silenced plants (41.9) than in the control (31.5) (Fig. 13A). It is well known that photosynthetic efficiency has a direct effect on the chlorophyll content^55^. To assess whether the knock-down of *CaDIR7* reduces the tolerance of pepper plants to salt and dehydration stress, leaf discs from TRV2:00 and *CaDIR7*-silenced plants were exposed to different concentrations (0, 100 and 300 mM) of NaCl and Mannitol. The results revealed that after 72 hours, the 0 mM-treated leaf discs had no significant differences in the chlorophyll content of both TRV2:00 and TRV2:*CaDIR7*, whereas in response to 100 and 300 mM NaCl and mannitol stresses, there was a significant decrease in the total chlorophyll content of TRV2:*CaDIR7* versus TRV2:00. Phenotypically, the leaf discs of TRV2:00 remained green and that of silenced plants exhibited a loss of chlorophyll (Fig. 13B and C). Intriguingly, as shown in Fig. 13D,E, the chlorophyll content of the *CaDIR7*-silenced plants leaf discs decreased drastically compared with TRV2:00 at the 300 mM concentration.

## Discussion

Dirigent proteins are an important disease resistance responsive multigene family in plants^29,56,57^. They play an important role in increasing resistance against stresses in different crops^11^. DIRs and their homologous have been described in all vascular plants^26^, and they are speculated to be involved with the production of lignin and lignan^26,58^. The number of DIR protein genes varies in different plant species. Previously, 25 DIRs in Arabidopsis, 19 DIRs in *I*. *indigotica*, 54 DIRs in rice, 35 DIRs in *Picea glauca* and 29 DIRs in *B*. *rapa* have been reported^9,11,27,29^. In the past, no study has been conducted on a genome-wide identification and characterization of DIR and DIR-like proteins in response to biotic and abiotic stresses in pepper. In the current study, we identified 24 CaDIRs for the first time in ‘CM334′ and ‘Zunla-1′ pepper genomes. Consistent with previous studies^9,11,27,29^, five well-conserved motifs were found in the amino acid sequence alignments of all 24 CaDIRs including *CaDIR7* (Fig. 1). The structural analysis showed that only 20.83% of CaDIRs contained one intron which contrasts with a previous study on the dirigent family in rice^56^. The ORFs analysis revealed that the amino acid (aa) sequences ranged from 106 aa (*CaDIR17*) to 330 aa (*CaDIR12*). The members of the DIR-e subfamily had an aa length longer than the CaDIRs from other subfamilies, ranging from 162 aa (*CaDIR19*) to 330 aa (*CaDIR12*). These results are supported by Li *et al*.^29^, who also found that the aa length of IiDIRs in the DIR-e subfamily was longer than in other subfamilies.

Previous studies have suggested that Asn sites are the features of secreted proteins observed in *FiDIR1*, the first and best characterized DIR protein^12,16^. In the present study, we found that 20 of the 24 CaDIRs had Asn sites, and 15 CaDIRs had more than one Asn site, which indicates that most of the CaDIRs seem to be secreted (Table 1). The X-ray or NMR structures of the dirigent proteins remain unavailable because DIRs have not yet been crystallized, and the tertiary structure of the DIRs are unknown^59,60^. Thus, to model DIRs, their homologous proteins with known structures can be used as templates^29,59^. Consequently, Phyre2 was used for the prediction of the tertiary structures as well as for the search for homologous proteins to CaDIRs. It was found that all of the CaDIRs are barrel-like proteins (Table 2). Meanwhile, Burlat *et al*.^16^ identified DIR genes and described their cellular localization and exclusive domain of lignification in *Forsythia intermedia* tissues. In the current study the subcellular location of all CaDIRs were predicted, and we found that pepper DIR proteins are targeted for extracellular release or possible final localization in the chloroplast, extracellular region, nucleus, cytoplasmic and vacuolar locations (Table 1).

The chromosomal locations revealed that CaDIRs were located on all 12 chromosomes of pepper, with chromosome 1 containing the highest number of CaDIRs (5). Similarly, the most BrDIRs (6) were found on chromosome A01 in *B*. *rapa*^9^. In the expansion of a gene family, tandem duplication, segmental duplication and transposition are the main evolutionary mechanisms^61^. Compared with tandem duplication and transposition, segmental duplication occurs frequently because of polyploidy in most plants, which conserve several duplicated chromosomal blocks in their genomes^61^. In the duplication analysis of CaDIRs, we obtained ten clusters of segmental duplication and two pairs of tandem duplication events (Table 3). In support of our study, tandem and segmental duplication events were also found in rice OsDIRs^56^. The results suggest that segmental and tandem duplications contribute to the expansion of the dirigent gene family in pepper.

Ralph *et al*.^27^ proposed that the DIR gene family can be grouped into six distinct subfamilies: DIR-a, DIR-b/d, DIR-c, DIR-e, DIR-f and DIR-g. Consistent with previous results from *I*. *indigotica*^29^, the 24 CaDIRs were separated into 3 subfamilies, DIR-a had 5 CaDIRs, DIR-b/d had 12 CaDIRs and DIR-e had 7 CaDIRs (Fig. 2A**)**. The members of the DIR-a subfamily are considered to be dirigent genes, involved in lignification and primarily associated with defense and resistance against pathogens and insects^13,27^. On the other hand, the biochemical functions of members of the DIR-b/d, DIR-c, DIR-f and DIR-g subfamilies are not known; thus, these genes are referred to as dirigent-like genes^11,27^. The present findings regarding CaDIRs belonging to the DIR-a, DIR-b/d and DIR-e subfamilies suggest that they share a common DIR ancestor and some similar biological functions^11,27,28^.

To further elucidate the potential roles of CaDIRs in the growth and development of pepper, the expression profiles of CaDIRs were examined in different tissues. The RT-qPCR analysis of the organ-specific expression showed that most of the CaDIRs exhibited the highest expression in flowers, followed by the stem, leaf, green fruit and red fruit, whereas the roots expressed the genes at the lowest levels of all organs. This is in contrast with previous studies on *B*. *rapa* and *I*. *indigotica*, where comparatively higher expression levels were observed in roots^9,29^; however, it is consistent, up to some extent, with the findings of Jin-long *et al*.^57^ and Damaj *et al*.^62^. In *F*. *intermedia*, the expression of DIR genes was also detected in different tissues^16^. In *B*. *hygrometrica*, it was anticipated that DIR proteins promote lignin biosynthesis, and the stem, root and flower buds were found to contribute to the biosynthesis of lignin^28^. Moreover, Rogers and Campbell^63^ also reported that lignification occurred throughout normal tissue development, and at specific sites lignin biosynthesis was induced by several biotic and abiotic stresses. The pepper CaDIRs were expressed in an organ-specific way, signifying the probable function of CaDIRs in lignin formation via specific organs and contribution to developmental processes.

In the development of vascular plants, lignin is crucial, and it mainly deposits in the vascular tissues; during plant development, it provides additional strength and imperviousness to the cell wall^64^. Moreover, lignin deposition in plants is considered a physical barrier against the penetration of pathogens^22^. Xu *et al*.^65^ also concluded that lignin had a critical role in the disease resistance of cotton. Among members of the *A*. *thaliana* DIR-a subfamily, *AtDIR5* and *AtDIR6* were different from other DIRs observed earlier^59,66^, and wherever *AtDIR6* was present, the final product of E-coniferyl alcohol was enantiomer (−)-pinoresinol. Later, Kim *et al*.^67^ confirmed that *AtDIR5* and *AtDIR6* engendered the preferential formation of (−)-pinoresinol. A recent study suggested that the dirigent protein family in plants contain several proteins involved in lignification and the response to pathogen infection as well as abiotic stresses^57^. In several plants, the changes in lignin content were observed to be caused by various biotic as well as abiotic stresses^23^. Lignin synthesis is a complex process because several gene families with many members are involved in the process^8^. Previous studies have reported changes in acid-soluble lignin content after biotic and abiotic stresses^8,9,28^. In the current study, the CaDIRs exhibited higher similarities with *A*. *thaliana*, indicating their involvement in the biosynthesis of lignin. In particular, members of the DIR-a subfamily showed identity based on topological tree (Fig. 3) and sequence alignments (Figure S1–S3) with *AtDIR5* and *AtDIR6*, which might have the ability to produce (−)-lignans. In light of this, we measured the acid-soluble lignin content of pepper plants inoculated with *P*. *capsici*. The results showed an increase after inoculation with the *P*. *capsici* strains; at 48 hpi, a greater-than-90% increase was noticed in the lignin content. Our results are supported by the findings of Zhang *et al*.^8^. After inoculation with *P*. *capsici*, most CaDIRs were also expressed at higher levels at 48 h, which further corroborate the acid-soluble lignin content results of the present study. Perhaps the CaDIRs enhanced the coupling of monolignols, thus increasing the lignin content of pepper plants. Taken together, these findings suggest that pepper CaDIRs might play a key role in the biosynthesis of lignin and consequently participate in the defense response of pepper.

Mostly, CaDIR expression was elevated with the inoculation of virulent and avirulent strains of *P*. *capsici*. Specifically, *CaDIR2*, *CaDIR3*, *CaDIR6*, *CaDIR7*, *CaDIR8*, *CaDIR11*, *CaDIR16*, *CaDIR22*, *CaDIR23* and *CaDIR24* showed significantly higher expression levels (Fig. 7). Moreover, the transcript levels of *CaDIR3*, *CaDIR16 and CaDIR24* were higher in plants inoculated with the avirulent strain versus the virulent strain, whereas *CaDIR2*, *CaDIR6*, *CaDIR7*, *CaDIR8*, *CaDIR11*, *CaDIR22* and *CaDIR23* had abundant transcript levels after inoculation with the virulent strain versus the avirulent strain. An earlier study also showed that two *G*. *barbadense* dirigent-like *Gbd1* and *Gbd2* genes were induced by a fungal disease *V*. *dabliae*^20^. The expression of the *CABPR1* gene in pepper was higher in the virulent strain interaction versus the avirulent interaction^68^. However, in our previous study^30^, it was found that the expression of most CaSBPs was comparatively higher in the avirulent interaction than in the compatible interaction. Meanwhile, other studies have revealed that the expression of oxysterol-binding protein gene (*CanOBP*) and a novel peroxidase gene (*CanPOD*) were higher in the incompatible interaction^49,69^. Some other defense-related genes such as b-1,3-glucanase gene (*CABGLU*), disease-associated protein gene (*CABPR1*), and peroxidase gene (*CAPO1*) were expressed in a similar pattern in the roots of pepper after inoculation with virulent and avirulent strains of *P*. *capsici*^70^. A phylogenetic analysis revealed that *CaDIR4* and *CaDIR14* showed a closer relationship with two *G*. *barbadense* genes (*Gbd1* and *Gbd2*), which were induced by *V*. *dahliae* infection^20^. Similarly, overexpression of the *GhDIR1* gene in cotton delayed the spread of the fungal pathogen *V*. *dahliae* in transgenic plants^64^. Moreover, *CaDIR6* and *CaDIR9* were adjacent to the *B*. *rapa* DIR-like gene (*BrDIR2*), which showed differential expression in *F*. *oxysporum* infection^9^. The differences in the expression patterns of CaDIRs and other defense-related genes might be due to the differences in the inoculation of *P*. *capsici* strains or the variation in their compatibility systems.

SA and MeJA intercede the responses of plants to biotic and abiotic stresses via a signal transduction pathway^71–73^. The basic defense to biotrophic pathogens is mediated by SA^72^, whereas it is controlled by MeJA in response to necrotrphic pathogens^74^. The cis-acting elements responsible for MeJA (TGACG-motif)^75^ and SA (TCA-element)^76^ were found in most of the CaDIRs promoters. In light of this, the pepper plants were exposed to SA and MeJA stresses in the current study to investigate their effects on the expression levels of representative CaDIRs (*CaDIR4*, *CaDIR7*, *CaDIR10*, *CaDIR12*, *CaDIR13*, *CaDIR14*, *CaDIR22* and *CaDIR23*) from each group. The qRT-PCR analysis confirmed that the expression of four CaDIRs was induced by MeJA and another four showed no significant response, whereas five CaDIRs were up-regulated by SA and three were down-regulated (Fig. 8). Our results corroborate the former studies on the inducibility of several DIR homologs. In *B*. *hygrometrica*, it was found that the expression of the *BhDIR1* gene was induced by SA^28^. Meanwhile, a study on *I*. *indigotica* reported that some liDIRs were up- and down-regulated by MeJA stress^29^. Similarly, Damaj *et al*.^62^ found that *SHDIR11* and *SHDIR16* were up-regulated by SA and MeJA. Moreover, the expression of four DIRs in sugarcane roots other than *SHDIR16*^77^, five DIRs in spruce stem^11^, and nineteen DIRs in the bark of the conifer *P*. *glauca*^27^ were also reported to be induced by MeJA treatment. The CaDIRs above were slightly expressed in the leaves under normal conditions but responded significantly to the hormonal treatments, especially *CaDIR7* and *23*, signifying their role in the defense response of pepper.

Earlier studies have shown that dirigent genes were expressed in different patterns in response to various abiotic stresses^9,28,56,78^. Hence, we extended our study to investigate the expression analysis of eight representative CaDIRs after salt (NaCl) and drought (mannitol) stresses (Fig. 8). The results revealed that five of these genes showed a significant response to one or both of these stresses. Wu *et al*.^28^ also reported a similar finding in one of their studies. Similarly, differential expression of 13 OsDIRs was noticed in rice after salt stress compared with mock-treated control seedlings^56^. Moreover, in sugarcane an increased *ScDIR* gene expression was reported in response to NaCl, PEG and oxidative stress treatments^57^. These findings show the potential roles of CaDIRs in the regulation of abiotic stress.

In phylogenetic analysis, the *CaDIR7* was clustered into DIR-e subfamily with six *A*. *thaliana* and seven *I*. *indigotica* genes (Fig. 3). *CaDIR7* existing on chromosome 3, having a 750 bp ORF, with MW of 26.09 kDa and *p*I 5.46, containing one intron, is predicted to localized in the nucleus (Table 1). *CaDIR7* also containing the Asn site, a feature of secreted proteins, indicating that it is likely to be a secreted protein (Table 1). The probability and identity analysis of *CaDIR7* homologous relationship revealed that it has 34% sequence identity with hypothetical protein c4revB belongs to disease resistance response protein 206 (drr206), while 21, 18 and 18% identity with AOC barrel-like proteins i.e. d2brja1, c4h69A and d1zvca1 respectively (Table 2). It has one segmental duplication (*CaDIR11*), while no tandem duplication was observed (Table 3). In conserved motif analysis of the CaDIR proteins through MEME server, it was found that *CaDIR7* containing the highest number of motifs (6) (Fig. 6). In its promoter region, a number of cis-acting elements including MeJA and SA responsiveness, defense and stress responsiveness, fungal elicitor-responsive element, heat stress responsiveness, ethylene-, auxin- and GA-responsive elements were found (Table 5). Besides *CaDIR7* showed the highest expression levels in response to *P*. *capsici*, MeJA, NaCl and mannitol stresses (Figs 7 and 8), while in tissue specific expression, it showed relatively higher transcription levels in stem and red fruit (Fig. 9). This further attracted our attention towards the importance of this gene. Thus, to affirm the role of *CaDIR7* against *P*. *capsici*, it was successfully knocked down in pepper plants through VIGS. An assay of the detached leaves of the *CaDIR7*-silenced and control plants with *P*. *capsici* inoculation was conducted. Larger lesions were observed in the leaves of silenced plants than in the control, indicating that silencing *CaDIR7* made the pepper leaves more prone to infection by *P*. *capsici*, following the same pattern as reported in our previous study^44^. The current finding was also supported by Ma and Liu^79^ on transgenic wheat lines. Our results are also consistent with findings that over-expression of the *GhDIR1* gene in cotton significantly delayed the invasion of the fungal pathogen *V*. *dahlia*^64^.

In addition, significant differences in *CaDIR7* expression levels were observed at 2, 4, 7 and 10 dpi in the control and silenced pepper plants. Wang *et al*.^80^ found that the root activity of pepper plants was higher with infection by the avirulent strain than by the virulent strain of *P*. *capsici*. In the current study on *P*. *capsici* pi, the root activity of the silenced plants decreased significantly compared with the control plants. This significant reduction in the root activity of the silenced pepper plants signifies severe injury to the roots caused by *P*. *capsici*. This concurs with our previous research work on pepper with a different gene *CaPTI1*^44^. Taken together, the larger leaf lesion areas and lessened root activity of the *CaDIR7*-silenced plants suggest the significant role of *CaDIR7* in the defense response of pepper against *P*. *capsici* infection.

In the present study, the leaf discs of *CaDIR7*-silenced plants showed evidently bleached phenotypes when exposed to salt and osmotic stresses compared with the control, whereas a significant decrease in chlorophyll content was observed in the silenced plants compared with the control. The REL assay in the current study revealed that after salt stress (300 mM), a significant increase in electrolyte leakage of the silenced plant leaf discs was recorded compared with the control. Consistent with our study, Chen *et al*.^81^ observed the same results for chlorophyll content after 3 days of salt and osmotic treatments, while electrical conductivity was significantly increased in *CaDHN1*-silenced plants after cold stress. Furthermore, the silenced and control plants exposed to NaCl stress (300 mM) showed a significant difference in the expression of *CaDIR7*. Furthermore, salt stress also altered the expression of the defense-related *CaDEF1* (a JA dependent) and *CaPO1* (a peroxidase) genes in the roots of silenced and control plants, indicating their role in salt stress tolerance. In response to high salinity, strong accumulation of the *CADEF1* transcript was observed in pepper^53^. A previous study also reported a marked increase in *CAPO1* mRNA levels after 4 days of treatment with copper in pepper stems, suggesting that this gene participates in the formation of defensive barriers^54^. More recently, *CaPO*2 transgenic *A*. *thaliana* plants exhibited more tolerance to high salt, drought, and oxidative stress^82^. Additionally, we measured the root activity after salt stress, and a significant reduction in the root activity of the silenced plants was observed compared with the control. This reduction in root activity is due to the stress injury caused by salt stress. Therefore, we speculate that *CaDIR7* is involved in the defense response of pepper against salt and drought stress.

## Electronic supplementary material

Supplementary Files

Supplementary Data set
