# Supplementary material for: Genome-wide analysis of dirigent gene family in pepper (Capsicum annuum L.) and characterization of CaDIR7 in biotic and abiotic stresses
Source: Sci Rep. 2018 Apr 3;8:5500. doi: 10.1038/s41598-018-23761-0 (PMC5883049; doi:10.1038/s41598-018-23761-0)
Supplement: Supplementary file 1 — Supplementary Files [file 41598_2018_23761_MOESM1_ESM.pdf]

Genome-wide analysis of dirigent gene family in pepper (*Capsicum annuum* L.) and characterization of *CaDIR7* in biotic and abiotic stresses

Abid Khan[1], Ru-Jian Li[1], Jian-Tian Sun[1], Fang Ma[1], Huai-Xia Zhang[1], Jing-Hao Jin[1], Muhammad Ali[1], Saeed ul Haq[1], Jun-E Wang[2] and Zhen-Hui Gong[1]

Supplementary Information

Supplementary Figure S1, S2 and S3. Alignment of CaDIRs sequences with some *A. thaliana* DIRs sequences.

Figure S1, Supplementary data

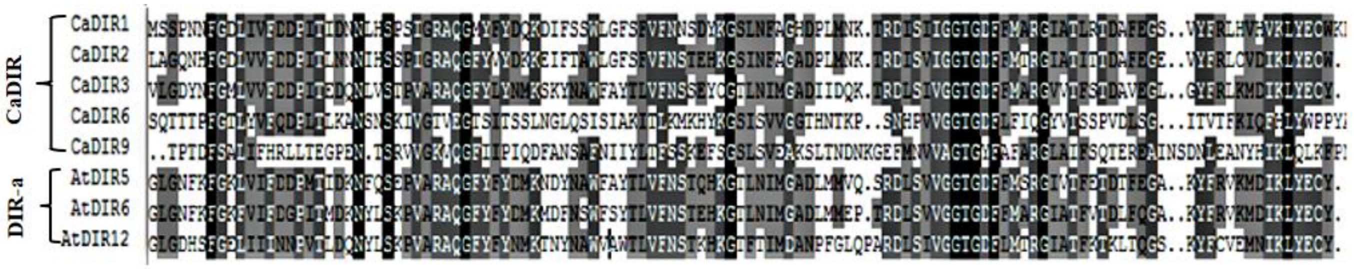

Figure S2, Supplementary data

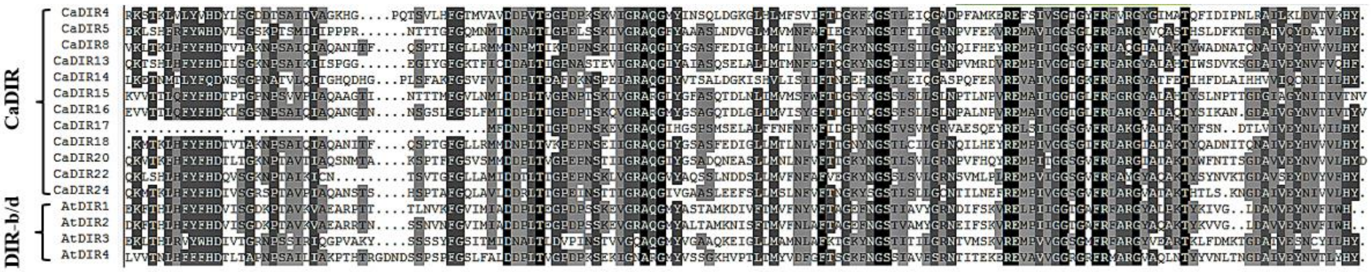

Figure S3, Supplementary data

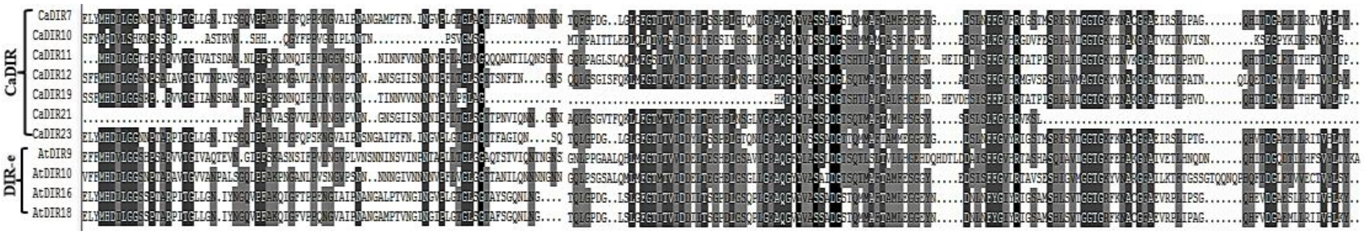

Table S1. Primers for gene sequencing and confirmation.

Gene Name	Accession#		Primer sequence (5'→3')
CaDIR1	CA01g01700	F	CGGTCCTTGTAAAGAAATGGTAT
		R	GCGTGATATCTAGAGTAATTTCC
CaDIR2	CA01g01710	F	CTTTATCAAAATGGCATCC
		R	ATAAAATTATTACCAGCACTCG
CaDIR3	CA01g01720	F	CGATTAAACCATGGAGAAATT
		R	GAAAATACAATTTAATAGCATTC
CaDIR4	CA01g15980	F	ATGGCTCTTTCTAATTCTTCTT
		R	TCAATAGTGCTTGACAGTGAC
CaDIR8	Capana04g001233	F	ATGGAAAATTTTTTGTTTATCGC
		R	TCAATAATGTAAAACCACAAC
CaDIR11	CA05g18990	F	CGTTCTCCTTTTTCATGCATGAC
		R	CGTGCTGTTTAAGGGGTGATG
CaDIR12	Capana06g000465	F	ATGGCATTTCAAAATTCCAATT
		R	CTAATAAGCAAGATACACAGTAA
CaDIR15	Capana08g002690	F	GGAATAGTTGTTCTCATTGTCG
		R	GTATCCAGCAATTCCATCACC
CaDIR16	CA08g18950	F	CTTCTTTCCTATGCACTACAG
		R	GAAGAAATCAAACATATGTGAC
CaDIR19	CA09g06650	F	ATGTTCTCCTCCTTCATGC
		R	CTAAGGCGTGAGGTAGAC
CaDIR21	CA11g13500	F	GTTGCAGGTACTGGTGTTGC
		R	GCAAGATGTGATTCAGAGACTC

Table S2. Primers for qRT-PCR of CaDIRs of pepper

Gene name	RT-PCR Primer sequence (5'→3')	Product length
CaDIR1	F: GGGAGTTTGAATTTTGCTGGAC<br>R: AGTAGCAATTCCTCTAGCCATG	105
CaDIR2	F: CGGCCCTGTAAAACCCTAAC<br>R: GTAAAATTTCCCCATGCTGGTGC	133
CaDIR3	F: CCATGACATCCTTTTCAACGTAAC<br>R: CATCCCAAAATTGTAATCTCCTAGG	103
CaDIR4	F: GAAGGTCCAGATCCAAAGTCC<br>R: TGAATCTCCAAAGTGCTCCC	143
CaDIR5	F: AGGGCACAAGGGTTTTACG<br>R: CTCTCACCTTCTCAAATACCGG	142
CaDIR6	F: CCCTAAAAGCCAACTCAAACTC<br>R: CTCCCACGACTGATATTGACC	147
CaDIR7	F: TCTTGACCTCGTCTCCTGAATTA<br>R: CACCTCCTTCAAACATAGCTG	126
CaDIR8	F: AGGGTAAGTATAATGGTAGCACAC<br>R: CAGCCCAATAAGTTTTCGCG	141
CaDIR9	F: ACTCCAACAGACTTTAGCGC<br>R: GATGAAACCTTGCACTTTCCC	96
CaDIR10	F: AAGACGGTAGCAGTCACATG<br>R: GCCCCTATGTACTCCGAAAAG	92
CaDIR11	F: CAGCCACACCAATTTCTCAC<br>R: TTGTCTCCAGTCCATCAGTTG	126
CaDIR12	F: AGTTTCAGGGGCAACTGGAG<br>R1: TCATCAGCACCAACACCAG<br>R2: ATCCTCCCAGGATATCGTGCATG	142
CaDIR13	F: ACAATGGGAGCGGCATAAG<br>R: CTCCACTCTTGACATCAGACC	147
CaDIR14	F: CTCTATTTTCAAGATTGGTCTGGTG<br>R: GGCAAAACTCAAAGGACCATG	87
CaDIR15	F: TTAATATGGGTGGCTGAAGGG<br>R: TCGGGACTACACTTGGATTTG	133
CaDIR16	F: CACTTACTATTGGACCTGACCC<br>R: AGGCTAAATGAACTCCCTTGG	148
CaDIR17	F: TTTTGTGTTCATTGATGGGCC<br>R: TCTATATTCCTGCGATTCAGCG	79
CaDIR18	F: GGCTTGATCTAAGTCCGAAGG<br>R: GCTGATGGATTCTTTGCAGTC	115
CaDIR19	F: CCAATAAATGTCGGAGTCCCTG<br>R: AGGCTAATGTATGGCTGGTG	87
CaDIR20	F: AGCCTTCTCATGAACCTCAAC<br>R: TGGTGAAATACAGGGTTCCG	92
CaDIR21	F: CAAGTGGAGTAGTTCTTGCAGTG<br>R: CGTTAGGAGTTGTCCCGCTAAGG	125
CaDIR22	F: ATGTTGCCACTTAGGGAGATG<br>R: ACTCAGAAACAGCATCACCAG	121
CaDIR23	F: ATCCAGAATAGCCAAACTAGTACTG<br>R: GTGCTTTCCCAAGGTTTTGTG	138
CaDIR24	F: GTACACTTAGTCTCCTTGGCTG<br>R: AGTCGGAAAACACCAGAACC	85

Table S3. Primers for gene silencing of *CaDIR7*.

Gene name	Primer sequence (5'→3')	Enzyme
*CaDIR7*VIGS-F	CCGGAATTCCGATTCTTGAACTGTACATGCAC	EcoRI
*CaDIR7*VIGS-R	CCGCTCGAGGCAAATATTGTGCCTGCTAGC	XhoI

Table S4. List of domains, formulas and total number of atoms of CaDIR and CaDIR-like genes of pepper.

No.	Name	Domain		Formula	Total number of atoms
		Dirigent	Transmembrane		
		start-end	start-end		
1	*CaDIR1*	1-146	---	$C_{800}H_{1175}N_{205}O_{228}S_{6}$	2414
2	*CaDIR2*	36-186	7--24	$C_{974}H_{1462}N_{250}O_{271}S_{7}$	2964
3	*CaDIR3*	35-182	4--26	$C_{957}H_{1463}N_{229}O_{272}S_{11}$	2932
4	*CaDIR4*	27-171	7--24	$C_{874}H_{1356}N_{222}O_{244}S_{6}$	2702
5	*CaDIR5*	46-188	---	$C_{959}H_{1485}N_{251}O_{269}S_{9}$	2973
6	*CaDIR6*	41-181	---	$C_{960}H_{1480}N_{252}O_{267}S_{4}$	2963
7	*CaDIR7*	105-248	---	$C_{1174}H_{1823}N_{309}O_{348}S_{8}$	3662
8	*CaDIR8*	44-188	---	$C_{981}H_{1513}N_{249}O_{269}S_{7}$	3019
9	*CaDIR9*	40-175	5--27	$C_{937}H_{1473}N_{239}O_{270}S_{3}$	2922
10	*CaDIR10*	47-228	10--32	$C_{1123}H_{1724}N_{300}O_{337}S_{10}$	3494
11	*CaDIR11*	78-233	---	$C_{1054}H_{1627}N_{295}O_{338}S_{2}$	3316
12	*CaDIR12*	182-329	13--30	$C_{1464}H_{2275}N_{389}O_{468}S_{7}$	4603
13	*CaDIR13*	47-189	7--29	$C_{959}H_{1466}N_{242}O_{272}S_{8}$	2947
14	*CaDIR14*	29-174	8--20	$C_{880}H_{1363}N_{229}O_{259}S_{3}$	2734
15	*CaDIR15*	36-180	---	$C_{885}H_{1392}N_{228}O_{257}S_{7}$	2769
16	*CaDIR16*	47-190	12--29	$C_{926}H_{1458}N_{246}O_{278}S_{7}$	2915
17	*CaDIR17*	1-104	---	$C_{525}H_{802}N_{134}O_{156}S_{3}$	1620
18	*CaDIR18*	42-186	---	$C_{945}H_{1475}N_{243}O_{267}S_{10}$	2940
19	*CaDIR19*	1-161	---	$C_{798}H_{1208}N_{216}O_{243}S_{2}$	2467
20	*CaDIR20*	44-188	---	$C_{964}H_{1496}N_{246}O_{274}S_{9}$	2989
21	*CaDIR21*	127-227	---	$C_{995}H_{1556}N_{274}O_{330}S_{3}$	3158
22	*CaDIR22*	8-147	---	$C_{740}H_{1133}N_{193}O_{217}S_{6}$	2289
23	*CaDIR23*	110-251	9--21	$C_{1179}H_{1858}N_{316}O_{359}S_{8}$	3720
24	*CaDIR24*	49-192	7--29	$C_{937}H_{1493}N_{253}O_{270}S_{7}$	2960

Table S6. Ten highly conserved motifs found in CaDIRs proteins.

No.	Motif #	Motif consensus sequence	E-value	Regular expression (RE) describing the motif.	Length (amino acids)
1	1	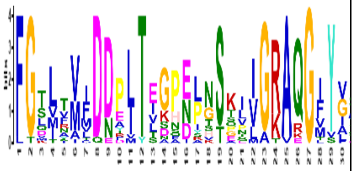	1.3e-274	FGTLT[VM][FI]DDP[LI]TEGP[EN][LP]NSKI[IV]G[RK]AQG[IF]Y[GV][AS][SA]SLD[GD]L[SG]LLM	41
2	2	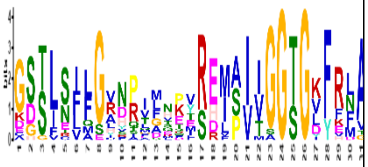	6.5e-269	G[SD][ST]L[SN][FI][LF]G[RV]N[PR]IXN[KP]V[RS]E[MI][ASP][IV][IV]GG[TS]G[KV]FR[LFN]ARG[YI]AT[AI]KTYX	41
3	3	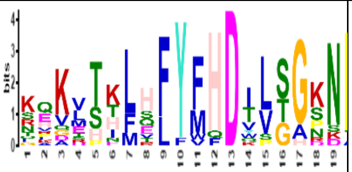	5.8e-110	K[QE]K[VL][TS][KHT][LF]H[FL]Y[FM]HD[IT][LV][SGT]G[KS]NP[ST]A[IRV][PQ]I[AT][QG]	27
4	4	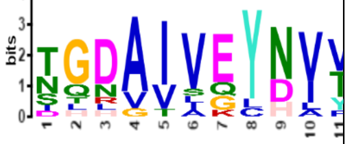	9.1e-035	[TN]GDA[IV]VEY[ND][VI][VT][VI]LHY	15
5	5	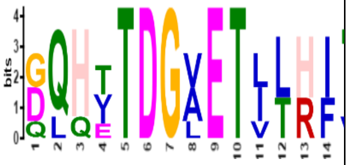	1.7e-017	[DGQ][QL][HQ][TEIV]TDG[AVL]ET[ILV][LT][HR][IF][TV]V[YH][LI][TA][YP]	20
6	6	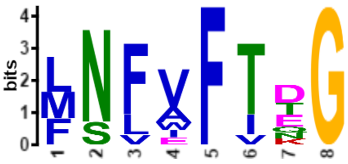	7.3e-017	[LFM][NS][FL][VA]F[TI][DET]GKY[NK]	11

**7**	**7**	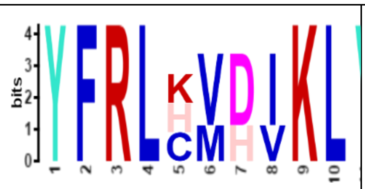	1.9e-013	YFRL[CHK][VM][DH][IV]KLYEC[WY]	14
**8**	**8**	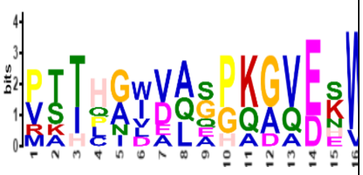	2.8e-013	[PV][TS][TI]H[GA][IW][VD][ALQ][GS][PG][KQ][GA][VQ][ED][KS]W[FA]K[AKR][LV][PD]	21
**9**	**9**	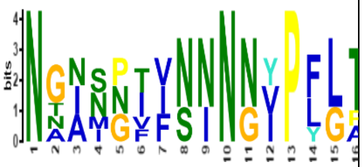	2.3e-006	NG[AIN][NS][GNP][IT][FIV][NS][NI]N[NG][IVY]P[FL][LG]TGL[AS]GT	21
**10**	**10**	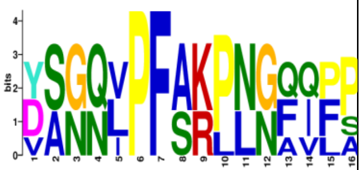	2.1e-005	[DYV][SA][GN][QN][LVI]PF[AS][KR][PL][NL][GN][FQA][IQV][FPL][PAS][IKV][ND][GNV][GV][VA]	21
